# Diversity of complementary diet and early food allergy risk

**DOI:** 10.1111/pai.70035

**Published:** 2025-01-27

**Authors:** Stina Bodén, Anna Lindam, Carina Venter, Richard Lundberg Ulfsdotter, Magnus Domellöf, Christina E. West

**Affiliations:** ^1^ Department of Clinical Sciences, Pediatrics Umeå University Umeå Sweden; ^2^ Department of Public Health and Clinical Medicine, Unit of Research, Education and Development – Östersund Umeå University Umeå Sweden; ^3^ Section of Allergy & Immunology, Department of Pediatrics, Children's Hospital Colorado University of Colorado School of Medicine Aurora Colorado USA; ^4^ Children's Hospital Colorado Aurora Colorado USA

**Keywords:** complementary foods, consumption frequency, diet diversity, food allergy prevention, infancy, NorthPop

## Abstract

**Introduction:**

Diet diversity (DD) in infancy may be protective for early food allergy (FA) but there is limited knowledge about how DD incorporating consumption frequency influences FA risk.

**Methods:**

Three measures of DD were investigated in 2060 infants at 6 and/or at 9 months of age within the NorthPop Birth Cohort Study: a weighted DD score based on intake frequency, the number of introduced foods, and the number of introduced allergenic foods. In multivariable logistic regression models based on directed acyclic graphs, associations to parentally reported physician‐diagnosed FA at age 9 and 18 months were estimated, including sensitivity and stratified analyses.

**Results:**

High weighted DD scores (24‐31p) at age 9 months were associated with 61% decreased odds of FA at age 18 months [OR (95% CI) = 0.39 0.18–0.88] compared with infants with the lowest DD scores (0‐17p). The association remained significant after exclusion of early FA cases. Having introduced 13–14 foods at age 9 months, independent of consumption frequency, was associated with 45% decreased odds of FA [OR (95% CI) = 0.55 (0.31–0.98)] compared to having introduced 0–10 foods. When stratifying, significantly reduced odds for FA were seen for children with eczema and for children with no FA history in the family. No association was seen between DD at age 6 months and FA at age 18 months.

**Conclusion:**

A diverse diet at age 9 months may prevent FA at age 18 months. Our results underscore the need for additional investigations on the impact of consumption frequency in infancy.

**Key message**

This study on food allergy risk, performed in the NorthPop Birth Cohort, Sweden, representing a general population, is the first to incorporate consumption frequency in measures of diet diversity in infancy. A diverse, complementary diet at around 9 months of age may reduce the risk of overall FA at 18 months of age, but a more diverse diet earlier than that does not seem to be protective. Children with a history of eczema may benefit the most from eating a diverse diet early in life for food allergy prevention.

## 1 INTRODUCTION

Food allergy (FA) is increasing globally^1^ and in Europe,^2^ including our region.^3^ Although the predisposition to allergy in general is inherited, infant diet can have major impact on the adaptive immune system and allergic disease development.^4^, ^5^, ^6^

Since 2019, the Swedish National Food Agency recommends to breastfeed the infant at least until 6 months of age and to introduce solid foods initially from around 6 months of age and from 4 months of age at the earliest, initially by small tastings and gradually serving larger portions from all food groups during the child's first year of life, irrespective of allergic heredity.^7^ We have shown that these updated complementary feeding guidelines have been positively implemented in families in the general population in Sweden; more types of food are served earlier to infants than before recommendations were updated, and the most significant change was seen for legumes and peanuts.^8^ There is high‐certainty evidence that early introduction of egg and peanut is associated with reduced egg and peanut allergy, respectively^9^; however, there is more uncertainty about timing and diversity of other allergenic foods.^9^, ^10^ The prevention of only two specific food allergies, that is, egg and peanut, may have limited public health impact, why it is desirable to investigate the impact of infant diet diversity (DD) and infant food allergen diversity on overall FA risk.^6^

Higher DD during the first year of life may be a preventive factor for FA and food sensitization in toddlers^11^, ^12^, ^13^ and in preschool and school age.^14^, ^15^, ^16^ Different measures of DD have previously been explored^12^ but to date, there is uncertainty whether the consumption frequency within a diverse diet can reduce overall FA risk.^15^ There is evidence that timing, dosage, interval, and regularity of allergen ingestion can all influence the development of oral tolerance.^17^, ^18^ RCTs have demonstrated that early regular oral exposure to food allergens will induce tolerance but previous studies on diet diversity have not incorporated consumption frequency in the risk assessment.

We sought to investigate different DD measures of complementary foods at 6 and 9 months of age, of which one of the investigated scores includes the frequency of food intake at 9 months, and estimate the adjusted associated risk to FA diagnosis at age 18 months, by using directed acyclic graphs (DAGs) based on a comprehensive literature review.^19^

## 2 METHODS

### 2.1 Study design

The NorthPop Birth Cohort Study (NorthPop) is an ongoing population‐based birth cohort study in the Västerbotten county in Northern Sweden where parents are invited to participate at the time of the routine ultrasound examination.^20^ The overall aim of NorthPop is to identify risk and protective factors for early onset non‐communicable diseases including allergy and asthma. In total, 10,000 pregnant women and their partners will be recruited, and the child will be primarily followed until 7 years of age. As of November 2024, over 9000 pregnant women and their partners have been recruited.

Inclusion criteria are pregnant women ≥18 years of age, comprehending the Swedish language, viable pregnancy at 14–24 weeks' gestation, and intention to give birth and reside in the catchment area in the next few years. Data on parental, fetal, and child health outcomes, lifestyle, diet, and environmental exposures are repeatedly collected using web‐based questionnaires during pregnancy and childhood until the children turn 7 years old. Biological samples including plasma, urine, feces, saliva, and breastmilk, are also collected.

**FIGURE 1:**
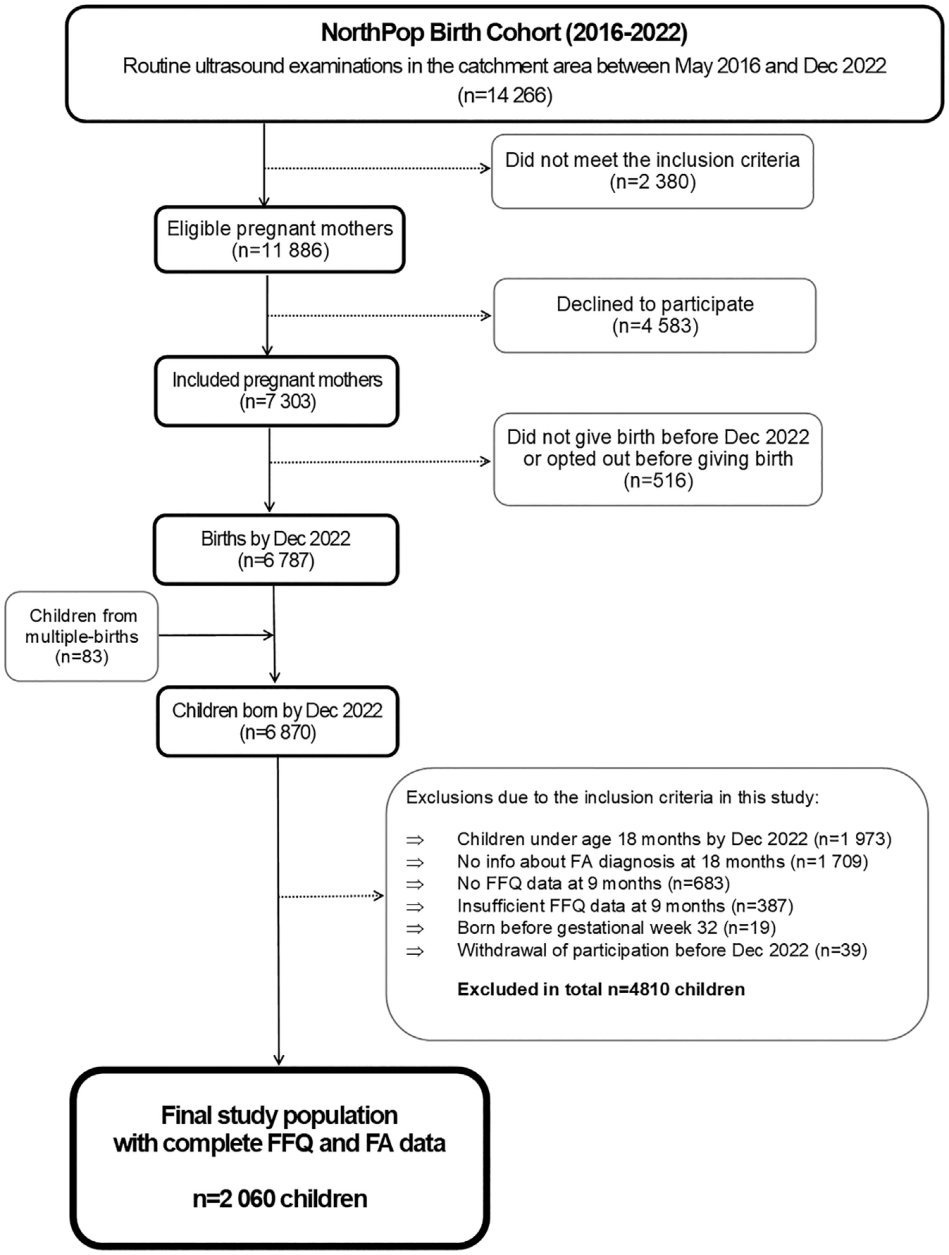
Flow chart of included and excluded participants. FA, food allergy; FFQ, food frequency questionnaire, N, number.

In the present study, families were recruited between May 2016 and December 2022, and data were prospectively collected from the time of recruitment until 18 months of age of the included children. Inclusion criteria in this study were to have sufficient dietary data collected at 9 months of age, to be at least 18 months old on December 31, 2022, and to have reported on physician‐diagnosed FA at 18 months of age. After exclusions, 2060 children were eligible. Details of inclusions and exclusions are found in the flowchart (Figure 1).

### 2.2 Ethics Statement

Ethical permission was granted by the regional Ethical Committee in Umeå, Sweden 2014/224–31, with amendments 2017–99‐32 M, 2018–175‐32 M, and 2018–504‐32. All parents were informed both verbally and in writing, and written informed consent was collected from both parents. All data handling complies with the European Union General Data Protection Regulation, and the study conforms with the Code of Ethics of the World Medical Association (Declaration of Helsinki, 1964).

### 2.3 Collection of dietary data

When the infants were around 9 months old, a food frequency questionnaire (FFQ) was sent to the parents. The FFQ included questions about the present consumption frequency of 55 food items on a scale from 1 to 6 where (1) Never, (2) 1–3 times/month, (3) 1–3 times/week, (4) 4–6 times/week, (5) 1–3 times/day, and (6) 4–6 times/day. The FFQ also included questions about timing of introduction of 41 food items with the following choices: (1) Not introduced at around 9 months of age, (2) Introduced at 0–3 months of age, (3) Introduced at 4–6 months of age, or (4) Introduced at 7 months of age or later. Portion size was not recorded.

### 2.4 Measurements of diet diversity

Measurements of DD were assessed based on the EAACI taskforce recommendations on how to measure DD in the context of allergy prevention (6) and on the updated Swedish infant feeding guidelines that recommend serving a variety of foods including frequent intake of fruit and vegetables, and irrespective of allergenic heredity, to serve legumes, nuts, and fish, as a part of the complementary diet.^7^ The three applied DD measurements are presented in Table 1: (1) A weighted DD score based on the frequency of consumption of 14 different foods at 9 months of age, (2) If 14 different foods had been introduced or not at 6 and 9 months of age, respectively, and (3) If 6 different allergenic foods had been introduced or not at 6 and 9 months of age, respectively.

**TABLE 1:** Measurements of diet diversity in infancy.

	Weighted diet diversity		Introduced foods	Introduced allergenic foods
Age at measurement	9 months		6 and 9 months	6 and 9 months
Points assigned	0‐2p/0‐3p for each food		Not introduced = 0p Introduced = 1p	Not introduced = 0p Introduced = 1p
Total range	0‐31p		0‐14p	0‐6p
Potatoes	x	0‐2p^a^	x	
Rice	x	0‐2p^b^	x	
Pasta	x	0‐2p^a^	x	
Bread	x	0‐2p^a^	x	
Porridge	x	0‐2p^a^	x	
Wheat				x
Egg	x	0‐2p^a^	x	x
Fish	x	0‐2p^a^	x	x
Meat	x	0‐2p^a^	x	
Dairy^c^	x	0‐2p^a^	x	x
Nuts and peanuts	x	0‐2p^a^	x	x
Fruit and berries	x	0‐3p^b^	x	
Vegetarian meat substitutions	x	0‐3p^b^		
Pulses	x	0‐2p^a^	x	
Vegetables	x	0‐3p^b^	x	
Soy			x	x

*Note*: The weighted diet diversity score was based on intake frequency of 14 foods, that is, how often the infant consumed each food at 9 months of age, based on present infant feeding guidelines. The other two measurements were based on if the infant had introduced 14 foods or not (yes/no), and 6 allergenic foods or not (yes/no), respectively. Both these diversity measurements were based on information about introduction of these foods at 6 and 9 months of age, respectively.

Abbreviation: p, points.

^a^ If not eaten at all = 0p, If eaten 1–3 times/month = 1p, if eaten 1–6 times/week = 2p, if eaten 1–6 times/day = 1 points. Note: 1 point assigned for certain foods consumed up to 6 times/day was based on the conception that more is not always better and thus not in line with what is considered to be a diverse diet.

^b^ If not eaten at all = 0p, if eaten 1–3 times/month = 1p, if eaten 1–6 times/week = 2p, if eaten 1–6 times/day = 3 points‐ Note: 3 points assigned for certain foods consumed up to 6 times/day was based on the recommendation to specifically eat plant‐based foods more frequently than other type of foods.

^c^ Any cows‐milk product excluding formula milk.

### 2.5 Covariates

Data on maternal characteristics; ethnicity (country of birth Sweden/other), University education (yes/no), smoking status (no smoking/smoking within a month before pregnancy/smoking during pregnancy), family history of FA, that is, mother and/or father and/or siblings (yes/no), and region/outdoor living environment (city/smaller community/urban) based on postal codes, were collected from web‐based questionnaires during pregnancy week 14–24.

Maternal body mass index (BMI) at registration at the maternity care center (MCC), Caesarean section delivery (yes/no), and if being a first‐time mother (yes/no) were collected from the Swedish Pregnancy Register.^21^ Maternal age at delivery (years) and gestational age (weeks) were retrieved from the NorthPop database. Child sex and birthweight in grams were retrieved from medical records.

The following data on infant feeding were parentally reported at age 4 months: type of feeding (exclusive breastfeeding/partial breastfeeding and formula feeding/exclusive formula feeding) at age 9 months: early introduction of complementary food (yes/no), that is, any kind of solid food introduced before the age of 4 months, and breastfeeding (yes/no).

### 2.6 Food allergy diagnosis at age 18 months

At ages 9 and 18 months, respectively, parents were asked if the child had a physician‐diagnosed FA at that age, if complete food elimination of the triggering food(s) at that age (yes/no) and type of symptom(s) together with age at onset of the food related symptom(s). Reported physician‐diagnosed FA at age 18 months was used as the main outcome in this study.

### 2.7 Statistical analysis

Analyses were conducted based on the complete case sample, as presented in Figure 1.

In a sensitivity analysis, we compared characteristics of the complete sample set of participants with excluded participants using chi‐square and *t*‐tests. We also compared mean values of each investigated DD scores between infants with and without early introduction of complementary food.

We used multivariable‐adjusted logistic regression models to assess potential associations between DD and FA, thus estimating the odds ratios (ORs) and 95% confidence intervals (CIs). Two adjusted models were assessed, and confounders were selected by using a Directed Acyclic Graph (DAG) based on a comprehensive literature review on modifiable risk and protective factors for childhood FA.^19^ As the review's working example was the effect of infant DD on FA, we directly used their online DAGitty‐code (http://dagitty.net/dags.html?id=8J8AWP). We identified four confounding factors that were included in multivariable model 1: breastfeeding status, early introduction of complementary food, maternal education level (used as a proxy for socioeconomic status), and maternal ethnicity (Figure S1). This first model can be regarded as the sufficient adjustment set with a minimal number of covariates that “must be controlled for”.^19^ In multivariable model 2, we added a set of confounders with evidence of causality according to the described literature review,^19^ namely maternal age at delivery, family history of FA, and outdoor living environment (Figure S1). The three exposure variables, all measuring DD, were assessed as both continuous variables and as categories constituting four groups for each exposure and based on the distribution of participants, where the first group of infants (=reference) had the lowest DD, whereas the fourth group of infants had the highest DD. We also assessed the FA risk at 9 months of age, and to control for reverse causality, we performed sensitivity analyses excluding early diagnosed infants.

Finally, since heredity of FA and infant eczema are known to be strong predictors for developing FA, stratified logistic regression models for children with vs. without a family history of FA and with vs. without history of eczema were conducted.

We made no adjustments for multiple comparisons because the hypothesis was made a priori and asked the same core question for all DD measurements. All analyses were conducted using IBM SPSS Statistics v29 (IBM Corp, Armonk, NY), and a *p*‐value of ≤.05 was considered statistically significant.

## 3 RESULTS

### 3.1 Descriptives

Characteristics of the 2060 included infants and their mothers are presented in Table 2. A comparison of characteristics of 4810 excluded and 2060 included participants are presented in Table S1. There were smaller differences regarding the infant's birthweight, gestational age, recruitment hospital, and regarding maternal characteristics including birth country, education level, history of FA, if first‐time mother, and age at delivery (Table S1).

**TABLE 2:** Characteristics of 2060 children and their mothers.

	*n* (%)/mean (SD)	Missing, *n* (%)
Child characteristics	2060	
Girls	1023 (49.7)	–
Caesarean section delivery	360 (17.5)	3 (0.1)
Gestational age (weeks, mean (SD))	39.4 (1.6)	–
Birthweight (grams, mean (SD))	3557 (509)	4 (0.0)
Type of feeding at 4 months of age		55 (2.7)
Exclusive breastfeeding	1339 (65.0)	
Partial breastfeeding	324 (15.7)	
Exclusive formula feeding	342 (16.6)	
Any breastfeeding at 9 months of age	809 (39.3)	65 (3.2)
Early introduction of solid foods (<4 months of age)	264 (12.8)	4 (0.2)
Diet diversity scores, mean (SD)^c^, ^d^		
Weighted diet diversity score at 9 months of age (range 0–31)	19.77 (3.91)	–
Diversity of introduced foods at 6 months of age (range 0–14)	8.99 (2.81)	4 (0.2)
Diversity of introduced foods at 9 months of age (range 0–14)	11.53 (1.51)	4 (0.2)
Diversity of introduced allergenic foods at 6 months of age (range 0–6)	2.94 (1.44)	4 (0.2)
Diversity of introduced allergenic foods at 9 months of age (range 0–6)	4.13 (1.06)	4 (0.2)
Adverse events from foods at age 9 months	271 (13.1)	1 (0.0)
FA diagnosis at age 9 months	73 (3.5)	1 (0.0)
FA diagnosis at age 18 months	100 (4.9)	–
Eczema by age 18 months	596 (28.9)	68 (3.3)
Family history of FA	564 (27.4)	26 (1.3)
Study Site		–
Umeå	1703 (82.7)	
Skellefteå	357 (17.3)	
Outdoor living environment^a^		–
City	1261 (61.2)	
Smaller Community	392 (19.0)	
Rural	407 (19.8)	
Maternal characteristics	2047^b^	
Country of birth other than Sweden	158 (7.7)	43 (2.1)
Maternal University education	1420 (68.4)	43 (2.1)
Maternal history of FA	363 (17.7)	46 (2.2)
BMI at registration at MCC, mean (SD)	25.00 (4.72)	45 (2.2)
Smoking status		28 (1.4)
No smoking	1959 (95.7)	
Smoked within a month before pregnancy	39 (1.9)	
Smoking in pregnancy	21 (1.0)	
Maternal age at delivery, years, mean (SD)	31.3 (4.3)	–
First‐time mother	1058 (51.7)	3 (0.1)

Abbreviations: BMI, body mass index; FA, food allergy; MCC, Maternity Care Center; n, number; SD, standard deviation.

^a^ Based on postal codes.

^b^ 2060 births, 13 births were twin births.

^c^ The distribution of each investigated DD score is presented in Figure S2.

^d^ A comparison of mean values for each DD score between infants that introduced solid foods early (*n* = 264) and those that did not (*n* = 1792) is presented in Table S2.

When comparing mean values of investigated DD scores between infants with and without having been introduced to solid foods before 4 months of age, there were significant differences in DD scores measured at age 6 months and for the weighted DD score measured at age 9 months (see Table S2).

### 3.2 Food allergy diagnosis

At 18 months of age, 100 children (4.9%) had a parentally reported FA diagnosis. Of these, 69 had a diagnosis of cow's milk allergy, 35 egg allergy, 7 wheat allergy, 7 peanut allergy, 4 soy allergy, 3 tree nut allergy, 1 fish allergy, and 30 had multiple FA at 18 months of age. Of the 100 children with parentally reported physician‐diagnosed FA, 98 had ingested the respective food allergen before diagnosis. Information on peanut introduction was missing for two children with reported peanut allergy. A study physician contacted these parents by telephone and reviewed medical records. Peanut introduction before the peanut allergy diagnosis could thus be confirmed in these two cases. In total, 57 reported specified symptoms of the eliciting foods; 37 had reacted with rashes, 1 with angioedema, 1 with asthma, 18 with vomiting, none with anaphylaxis, and 33 reported gastrointestinal symptoms, predominantly stomach pain, obstipation, diarrhea, and bloody stools.

Mean (SD) age at onset of those who reported symptoms to food at age 9 months was 3.05 (SD 2.07) months. At 18 months of age, mean (SD) age of onset in months was reported related to each specific ingested food: milk 2.81 (2.25), egg 7.22 (3.32), nuts 11.80 (5.35), peanut 11.00 (5.35), peas 10.33 (4.04), soy 6.50 (1.29), wheat 7.67 (4.68), fruit 8.29 (4.03), and adverse reaction to other foods 10.56 (3.81).

Among children whose parents reported no FA diagnosis at age 18 months (*n* = 1960), the majority had been introduced to 4–6 allergens by that age (98.9%), see Table S3. Children whose parents had reported no FA and not having introduced all six allergens by age 18 months, were regarded as non‐allergic.

After multivariable adjustments in models 1 and 2, a total of 92 children with FA were included in the analyses.

### 3.3 Diversity of complementary feeding and food allergy risk

#### 3.3.1 Weighted diet diversity scores at age 9 months

The higher the weighted DD score at 9 months of age, the lower was the associated odds of having FA at 18 months of age, although not statistically significant (OR per 1 unit increase in multivariable model 2: 0.96, 95% CI 0.91–1.01), Table 3. However, for the group of infants with the highest DD scores (24–31 points), there was a statistically significant association to 61% decreased odds of FA compared with the lowest DD scores (0–10 points), after full adjustment (OR multivariable model 2: 0.39, 95% CI: 0.18–0.88, Table 3).

**TABLE 3:** Diet diversity of complementary feeding in infancy and the association to food allergy at 18 months of age.

	Unadjusted Model				Multivariable Model 1^d^				Multivariable Model 2^e^			
	*n*	FA cases	OR (95% CI)	*p*	*n*	FA cases	OR (95% CI)	*p*	*n*	FA cases	OR (95% CI)	*p*
Weighted diet diversity score at age 9 months^a^												
Continuous, 0‐31p	2060	100 (4.9)	0.97 (0.92–1.02)	.236	1960	92 (4.7)	0.97 (0.92–1.02)	.182	1957	92 (4.7)	0.96 (0.91–1.01)	.128
0‐17p	494	28 (5.7)	Ref 1.0		469	28 (5.9)	Ref 1.0	Ref	469	28 (5.9)	Ref 1.0	Ref
18‐20p	654	34 (5.2)	0.91 (0.55–1.53)	.913	622	29 (4.7)	0.78 (0.46–1.35)	.787	620	29 (4.7)	0.78 (0.45–1.34)	.362
21‐23p	582	29 (5.0)	0.87 (0.51–1.49)	.617	552	27 (4.9)	0.84 (0.48–1.45)	.837	552	27 (4.9)	0.80 (0.46–1.38)	.416
24‐31p	330	9 (2.7)	0.47 (0.22–1.00)	.051	317	8 (2.5)	0.42 (0.19–0.94)	.035	316	8 (2.5)	0.39 (0.18–0.88)	.023
Diversity of introduced foods at age 6 months^b^												
Continuous, 0–14 foods	2056	100 (4.9)	1.02 (0.95–1.09)	.661	1960	92 (4.7)	1.00 (0.93–1.09)	.901	1957	92/1957	0.99 (0.92–1.08)	.885
0–7 foods	512	24 (4.7)	Ref 1.0		485	23 (4.7)	Ref 1.0		485	23 (4.7)	Ref 1.0	
8–9 foods	563	24 (4.3)	0.91 (0.51–1.62)	.736	535	21 (3.9)	0.84 (0.46–1.54)	.569	534	21 (3.9)	0.81 (0.44–1.50)	.505
10–11 foods	618	32 (5.2)	1.11 (0.65–1.92)	.705	597	31 (5.2)	1.07 (0.61–1.87	.826	596	31 (5.3)	1.02 (0.58–1.80	.946
12–14 foods	363	20 (5.5)	1.19 (0.65–2.18)	.584	343	17 (5.0)	1.07 (0.55–2.06)	.842	342	17 (5.0)	0.99 (0.51–1.91)	.966
Diversity of introduced foods at age 9 months^b^												
Continuous, 0–14 foods	2056	100 (4.9)	0.89 (0.79–1.01)	.072	1960	92 (4.7)	0.89 (0.78–1.02)	.098	1957	92 (4.7)	0.88 (0.77–1.01)	.065
0–10 foods	465	32 (6.9)	Ref 1.0		439	29 (6.6)	Ref 1.0		438	29 (6.6)	Ref 1.0	
11 foods	490	21 (4.3)	0.61 (0.34–1.07)	.083	465	20 (4.3)	0.61 (0.34–1.09)	.094	464	20 (4.3)	0.58 (0.32–1.06)	.075
12 foods	519	24 (4.6)	0.66 (0.38–1.13)	.129	495	21 (4.2)	0.64 (0.36–1.14)	.131	495	21 (4.2)	0.61 (0.34–1.10)	.100
13–14 foods	582	23 (4.0)	0.56 (0.32–0.97)	.037	561	22 (3.9)	0.59 (0.32–1.05)	.073	560	22 (3.9)	0.55 (0.31–0.98)	.043
Diversity of allergenic foods introduced at age 6 months^c^												
Continuous, 0–6 allergenic foods	2056	100 (4.9)	1.06 (0.93–1.22)	.382	1960	92 (4.7)	1.05 (0.91–1.23)	.490	1957	92 (4.7)	1.03 (0.89–1.21)	.667
0–2 allergenic foods	777	29 (3.7)	Ref 1.0		738	27 (3.7)	Ref 1.0		738	27 (3.7)	Ref 1.0	
3 allergenic foods	541	34 (6.3)		.035	513	31 (6.0)	1.63 (0.95–2.77)	.074	512	31 (6.1)	1.57 (0.42–2.93)	.099
4 allergenic foods	442	24 (5.4)	1.48 (0.85–2.58)	.165	426	22 (5.2)	1.42 (0.79–2.54)	.239	425	23 (5.4)	1.34 (0.74–2.41)	.331
5–6 allergenic foods	296	13 (4.4)	1.19 (0.61–2.31)	.619	283	12 (4.2)	1.18 (0.58–2.39)	.642	282	13 (4.6)	1.10 (0.54–2.43)	.791
Diversity of allergenic foods introduced at age 9 months^c^												
Continuous, 0–6 allergenic foods	2056	100 (4.9)	0.91 (0.75–1.10)	.314	1960	92 (4.7)	0.93 (0.76–1.13)	.460	1957	92 (4.7)	0.91 (0.75–1.12)	.371
0–3 allergenic foods	548	29 (5.3)	Ref 1.0		518	27 (5.2)	Ref 1.0		517	28 (5.2)	Ref 1.0	
4 allergenic foods	741	38 (5.1)	0.97 (0.59–1.59)	.896	703	33 (4.7)	0.89 (0.53–1.50)	.664	702	33 (4.7)	0.89 (0.56–1.50)	.652
5 allergenic foods	575	28 (4.9)	0.92 (0.54–1.56)	.747	554	27 (4.9)	0.98 (0.56–1.69)	.931	554	27 (4.9)	0.94 (0.54–1.83)	.820
6 allergenic foods	192	5 (2.6)	0.48 (0.18–1.25)	.134	185	5 (2.7)	0.53 (0.20–1.41	.205	184	5 (2.7)	0.50 (0.19–1.33)	.164

Abbreviaions: CI, confidence interval; DD, diet diversity; FA, food allergy; N, number; OR, odds ratio.

^a^ Based on consumption frequency of 14 different food groups, each group contributing with 0–2 or 0–3 points to the total score.

^b^ Based on introduction (yes/no) of 14 different food groups (potatoes, rice, pasta, vegetables, meat, fish, soy, legumes, fruit and berries, egg, dairy, porridge, bread, nuts & peanuts).

^c^ Based on introduction (yes/no) of 6 different allergenic foods: milk, wheat, egg, fish, soy, nuts & peanuts.

^d^ Adjusted for ethnicity (Swedish or other), maternal University education (yes/no), breastfeeding status at 4 months (exclusively breastmilk, breastmilk and formula, or only formula), and early (<4 months) introduction of any solid food (yes/no).

^e^ Adjusted for ethnicity (Swedish or other), maternal University education (yes/no), breastfeeding status at 4 months (exclusively breastmilk, breastmilk and formula, or only formula), early (<4 months) introduction of any solid food (yes/no), FA in the family (mother, and/or father, and/or sibling), housing outdoor environment (city, smaller village, or rural), and maternal age at delivery.

There was no association between DD scores and FA as early as 9 months of age (Table S3) but in sensitivity analysis for the FA risk at age 18 months, excluding 48 infants with a FA diagnosis at age 9 months, results remained significant for the weighted DD score (OR multivariable model 2: 0.24, 95% CI: 0.07–0.83, Table S4), although limited by power.

#### 3.3.2 Diversity of foods introduced at age 6 and 9 months

For diversity of introduced foods at 6 months of age, unadjusted OR was above 1 and hence did not indicate any association to reduced subsequent FA risk (Table 3). In the fully adjusted multivariable model 2, OR for 13–14 introduced foods compared to 0–7 introduced foods was 0.99, 95% CI 0.51–1.91 (Table 3).

For complementary diet at 9 months of age, results demonstrated an association between infants with the highest number of introduced foods (13–14 foods) and 45% decreased odds of FA (OR multivariable model 2: 0.55, 95% CI: 0.31–0.98) compared to infants having introduced a limited number of complementary foods (0–10 foods); see Table 3.

No association was seen between the diversity of introduced foods at 6 and 9 months and FA at 9 months of age (Table S4). When excluding infants with early FA diagnosis (*n* = 48), the OR of 0.55 remained for the group that consumed the highest number of different foods (13–14 foods) at 9 months of age compared to infants that had only introduced up to 10 different foods, but did not reach statistical significance (Table S5).

#### 3.3.3 Diversity of allergenic foods introduced at age 6 and 9 months

Having introduced more allergenic foods at 6 and 9 months of age, respectively, was not associated with FA risk at age 18 months OR for 5–6 allergenic foods at 6 months age in multivariable model 2: 1.10, 95% CI 0.54–2.43 and OR for consuming 6 allergenic foods at 9 months age in multivariable model 2: 0.50, 95% CI 0.19–1.33 (Table 3), nor when early FA cases were excluded (Table S5). Weighted food allergen DD also did not show any association with FA at 18 months (data not shown).

### 3.4 Sensitivity analysis excluding children with gastrointestinal symptoms

To reduce the risk of diagnostic misclassification, 33 children with FA at age 18 months reporting only gastrointestinal symptoms were excluded, in which results for the highest DD measured at age 9 months, including both the weighted DD score and the number of introduced foods, remained significant (Table S6).

### 3.5 Predictors of food allergy at 18 months of age and stratification analysis

In fully adjusted models based on the applied DAG model (Figure S1), two factors were statistically significantly associated with a higher FA risk, namely if the child was fed both breastmilk and formula (OR 1.77 and 95% CI 1.03–3.05) or exclusively formula fed (OR 1.89 and 95% CI 1.12–3.18) compared to being exclusively breastfed at age 4 months, and if having FA in the closest family (OR 2.51 and 95% CI 1.64–3.84), Table S7. The other covariates included in multivariable model 2 were not significantly associated with FA risk, including early introduction of solid foods, that is, before 4 months of age (Table S7).

**FIGURE 2:**
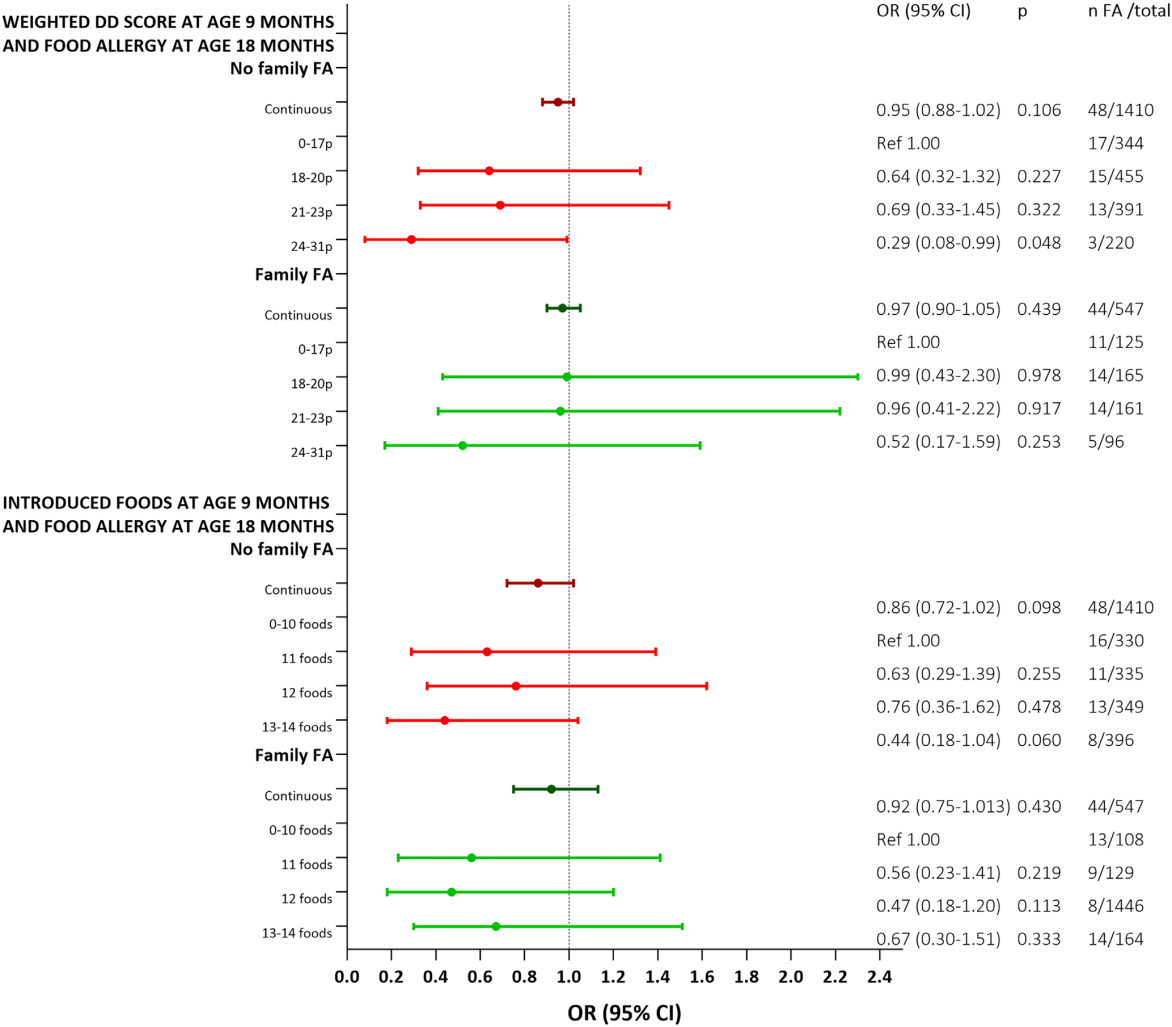
Forest plots representing odds ratios (ORs) and 95% CIs for the association between two measures of diet diversity at 9 months of age and of food allergy (FA) at 18 months of age, stratifying for infants without versus with a history of FA in the closest family (mother and/or father and/or sibling). Forest plots and numbers represents the fully adjusted model (Model 2), adjusted for ethnicity (Swedish or other), maternal University education (yes/no), breastfeeding status at 4 months (exclusively breastmilk, breastmilk and formula, or only formula), early (<4 months age) introduction of any solid foods (yes/no), outdoor living environment (city, smaller village, or rural), and maternal age at delivery. Estimates from all unadjusted and adjusted models are presented in Table S7. CI, confidence interval; DD, diet diversity; FA, food allergy; OR, odds ratio; Ref, reference.

When stratifying for family history of FA, a potential reduction in FA risk was seen for children that had no FA in the family and reported the highest weighted DD scores at 9 months of age (OR 0.29 and 95% CI 0.08–0.99), demonstrated with forest plots for the fully adjusted model in Figure 2 with all models presented in Table S8.

**FIGURE 3:**
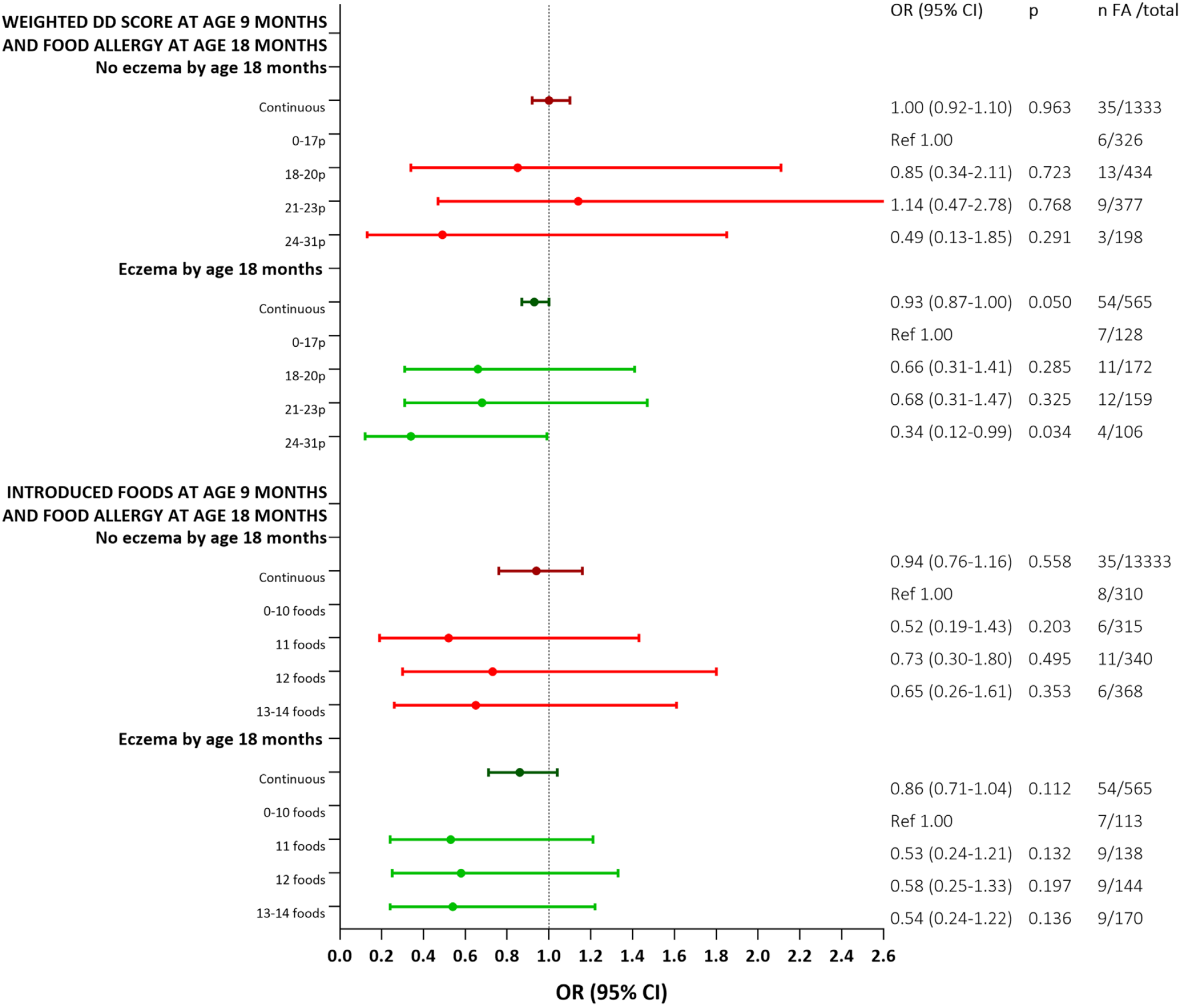
Forest plots representing odds ratios (ORs) and 95% CIs for the association between two measures of diet diversity at 9 months of age and food allergy (FA) at 18 months of age, stratifying for infants without vs. with a history of eczema up until 18 months of age. Forest plots and numbers represent the fully adjusted model (Model 2), adjusted for ethnicity (Swedish or other), maternal University education (yes/no), breastfeeding status at 4 months (exclusively breastmilk, breastmilk and formula, or only formula), early (<4 months age) introduction of any solid foods (yes/no), outdoor living environment (city, smaller village, or rural), and maternal age at delivery. Estimates from all unadjusted and adjusted models are presented in Table S8. CI, confidence interval; DD, diet diversity; FA, food allergy; OR, odds ratio; Ref, reference.

Interestingly, when stratifying for history of eczema by 18 months of age, significant associations were particularly seen between the weighted DD score and FA at age 18 months for children with a history of eczema (OR per 1 unit increase in multivariable model 2: 0.93, 95% CI 0.87–1.00), see Figure 3 and Table S9.

## 4 DISCUSSION

In this prospective population‐based birth cohort study, a high weighted DD score based on the novel approach to calculate consumption frequency of complementary foods already at 9 months of age was associated with a decreased risk of FA at 18 months of age, particularly for children with a history of eczema and children with no family history of FA. Having introduced a larger number of complementary foods at 9 months of age, irrespective of consumption frequency, was also indicative of a reduced FA risk, whereas this was not true for having introduced more complementary foods as early as at 6 months of age. Diversity of typical allergenic foods introduced at ages 6 and 9 months, respectively, was not associated with lower overall FA.

Two recent publications have underscored that studies examining the role of consumption frequency of foods in the risk reduction of FA are highly requested.^4^, ^15^ It is evident that repeated exposure to foods in infancy is important both for tolerance development and maintenance.^22^ From our results using the weighted DD score, one could assume that frequent consumption of plant‐based foods including fruit, vegetables, and legumes which are rich in fiber and vitamins are especially important for reducing FA risk, in line with existing guidelines.^7^, ^8^ Among former observational studies reporting associations between infant DD and FA risk, none calculated the frequency or amounts of consumed foods^11^, ^12^, ^14^, ^15^, ^16^, ^23^ and the novel result in the present study calls for more investigations on intake frequency for FA prevention.

The Swedish updated guidelines on complementary feeding do not advice against introducing diverse foods before the age of 6 months but also do not recommend to introduce diverse foods before that age,^7^ in line with European guidelines.^24^ The null association between DD at 6 months of age and FA risk could be caused by a generally low DD in the study population at that early age, whereas at 9 months of age, measures of DD are more distinguishable and hence, in line with existing guidelines.^7^ Early introduction is a highly discussed topic,^25^ and it should be noted that introduction of solid foods before 4 months of age was per se not associated to reduced FA risk in our multivariable adjusted models. However, comparative mean values of DD scores for early vs. non‐early introduction, indicated that it might be more likely to achieve a diverse diet at age 6 months and for the weighted score, also at age 9 months, if solid foods are introduced before the age of 4 months. This is an important message that should be addressed by health care providers and groups responsible for nutritional guidelines.

Further, our results are consistent with previous studies that found stronger associations to DD later in infancy, that is, from the age of 8 months.^11^, ^12^, ^15^ In the Enquiring About Tolerance (EAT) study, only 42% in the intervention group could adhere to the multiple food regimen, demonstrating the challenge to introduce multiple foods as compared to single or fewer food items in early infancy.^26^ To achieve a high DD before the age of 6 months, a wide range of foods is probably necessary to introduce already at around 4 months of age, for which convincing benefits on overall FA prevention is still lacking.^24^, ^27^ Hence, the overall picture is that a high DD is most likely not necessary to achieve as early as at 6 months of age but clearly appears beneficial in later infancy.

Recent infant feeding guidelines advise against delaying the introduction of typical allergenic solid foods, although certainty of evidence can be regarded as low.^7^, ^24^, ^27^, ^28^ Based on the null association for diversity of typical allergenic foods consumed at 6 and 9 months of age in this study, no further strength of evidence can be added to the hypothesis of diverse allergenic foods being protective. Nor can it be shown in the present study that diversity of allergenic foods increases the risk of FA. Hence, we are still limited to draw conclusions about the preventive meaning of a diverse introduction of typical allergenic foods in specific, but based on former studies including RCTs, the total impression is that no harm is caused from introducing multiple food allergens in early infancy.^28^, ^29^ Likely, it is more important to cover an overall diverse diet in infant feeding regimes. Studies using 16 S rRNA sequencing have shown distinct differences in gut microbiota composition between children with and without FA^30^ and in children with allergic comorbidity.^31^ Complementary diet largely affects the gut microbiota after the weaning period around 9–18 months of age where this window of opportunity has shown a distinct shift in gut microbial composition.^32^ Hence, a more diverse diet during this critical period of early life may have significant immune‐modulating effects, operating through the gut microbiota.^33^

We also addressed the impact of atopy and allergic heredity by stratifying for history of eczema in the child and for FA in the family, respectively. For children whit a history of eczema, there was a potential protective association to FA if a more diverse diet was reported at age 9 months. This is an interesting result, strengthening the dual allergen exposure hypothesis with regard to early oral multiple food exposure.^34^ In the absence of FA heredity, there was also a potential reduction in associated risk for infants eating the most diverse diet at 9 months of age, even if somewhat limited by power. This is an important indication, and although it has been shown that high‐risk infants could gain from a diverse diet with regard to egg allergy^16^ and from early introduction of peanuts with regard to peanut allergy,^35^ an overall protective effect on FA from high DD in high‐risk infants cannot be stated based on this stratified analysis.

Major strengths of this study are the population‐based design reducing the risk of selection bias, the extensive collection of data, the access to high‐quality national register data, the direct use of the literature‐based DAGs^19^ risk analyses chosen a priori, which enable future comparison with studies applying the same DAG model. In addition, the use of food frequency data in infancy adds meaningful insights about the relation to FA prevention in a general population.

There are also limitations including the proportion of missing values that reduces statistical power and increases the risk of selection bias; smaller differences in characteristics of excluded and included participants were detected. Children not exposed to all six common food allergens by age 18 months were treated as non‐allergic which may be subject to misclassification. We relied on self‐reported data on FA prevalence but the number of self‐reported FA was not considered overestimated in relation to European figures of a 4%–6% IgE‐mediated FA prevalence in children.^7^ On the other hand, the use of physician‐diagnosed FA may lead to an underestimation of the true prevalence but reduces the risk of detection bias. Lack of repeated measures of food intake in infancy, and insufficient follow‐up data on FA incidence in the study population to date are other limitations. However, as NorthPop is still under recruitment, we predict great potential in conducting follow‐up analyses in the forthcoming years.

In conclusion, a diverse diet at 9 months of age may reduce the risk of overall FA at 18 months of age in a general population, predominantly in children with a history of eczema and children without positive heredity for FA. The results support existing infant feeding guidelines for early FA prevention since introducing a diverse diet after 6 months of age appears to be safe and may prevent FA.

## AUTHOR CONTRIBUTIONS

**Stina Bodén:** Conceptualization; methodology; formal analysis; writing – review and editing; visualization; writing – original draft; data curation; investigation; funding acquisition. **Anna Lindam:** Methodology; writing – review and editing; formal analysis. **Carina Venter:** Methodology; writing – review and editing. **Richard Lundberg Ulfsdotter:** Data curation; writing – review and editing; resources; project administration. **Magnus Domellöf:** Resources; writing – review and editing; funding acquisition. **Christina E. West:** Conceptualization; resources; supervision; writing – review and editing; investigation; funding acquisition; methodology.

## FUNDING INFORMATION

This study was funded by the Swedish Research Council grant number 2018‐02642(CEW); the Heart‐Lung Foundation grant number 20180641 (CEW); the Ekhaga Foundation grant number 2018–40 (CEW); the Västerbotten County Council (ALF) grant numbers RV 832441, RV 840681 (CEW), and RV‐960756 (SB), Umeå University's strategic research funds (SB), and the Unit of Research, Region Jämtland Härjedalen Foundation grant number JLL‐993235 (SB) and grant number JLL‐993810 (SB). The NorthPop infrastructure receives funding from Västerbotten County Council and Umeå University (CEW and MD). The funding bodies had no role in study design, data collection and analysis nor in the preparation of the manuscript.

## CONFLICT OF INTEREST STATEMENT

Dr. Venter reports grants from Reckitt Benckiser Food Allergy Research and Education, National Peanut Board; personal fees from Reckitt Benckiser, Nestle Nutrition Institute, Danone, Abbott Nutrition, Else Nutrition, Before Brands, and Owen outside the submitted work. Dr. West has received research funding from Thermo Fisher Scientific and Arla, which was directly paid to the institution, and speaker honorarium from Thermo Fisher Scientific, Aimmune Therapeutics, a Nestlé Health Science company, and Nutricia outside the submitted work. The other authors declare no conflicts of interest.

### PEER REVIEW

The peer review history for this article is available at https://www.webofscience.com/api/gateway/wos/peer‐review/10.1111/pai.70035.

## Supporting information


Appendix S1.
