# Supplementary material for: Diversity of complementary diet and early food allergy risk
Source: Pediatr Allergy Immunol. 2025 Jan 27;36(1):e70035. doi: 10.1111/pai.70035 (PMC11771557; doi:10.1111/pai.70035)
Supplement: Supplementary file 1 — Appendix S1. [file PAI-36-e70035-s001.docx]

| Description of supplementary material to the study Diversity of complementary diet and early food allergy risk | Page |
| --- | --- |
| Suppl T1. Characteristics of 2060 included children and 4810 excluded children and their mothers. | 2 |
| Suppl T2. Comparison of mean (SD) values for each investigated diet diversity score between the group of infants that reported early introduction (<4 months of age) to solid foods (n=264) and the group that had not introduced solid foods before the age of 4 months (n=1792). | 3 |
| Suppl T3. Food allergen exposure by age 18 months among children with no parentally reported food allergy by that age (N=1960) | 3 |
| Suppl T4. Different measures of diet diversity of complementary feeding in infancy and the association to food allergy at age 9 months presenting unadjusted and multivariable adjusted odds ratios (ORs) and 95% Confidence Intervals (CIs), together with P-values. | 4 |
| Suppl T5. Sensitivity analysis for associations between diet diversity of complementary feeding in infancy and food allergy at age 18 months, excluding children with a food allergy diagnosis already at age 9 months (n=48). | 5-6 |
| Suppl T6. Sensitivity analysis for the association between diet diversity of complementary feeding in infancy and food allergy (FA) at age 18 months, excluding children with reported gastrointestinal symptoms as response to their FA (n=33). | 7-8 |
| Suppl T7. Risk estimates from the multivariable logistic regression model 2 for the association between different measures of diet diversity of complementary foods at 6- and 9-months age and the risk of food allergy (FA) at age 18 months. | 9-10 |
| Suppl T8. Stratified analyses of children without vs with a history of food allergy in the closest family (mother and/or father and/or sibling), estimating unadjusted and adjusted associated risk for the child developing their own food allergy at age 18 months in relation to two measurements of diet diversity at age 9 months (weighted diet diversity score and diversity of introduced foods). | 11-12 |
| Suppl T9. Stratified analyses of children without vs with a history of eczema up until 18 months of age, estimating unadjusted and adjusted associated odds for the child developing food allergy at age 18 months in relation to two measurements of diet diversity at 9 months of age (weighted diet diversity and diversity of introduced foods). | 13 |
| Suppl Fig 1. Directed Acyclic Graph used for identification and selection of confounding factors used in multivariable model 1 and 2 for estimating associations between diet diversity and food allergy risk. | 14-15 |
| Suppl Fig 2. Distribution and mean values (SD) of investigated diet diversity (DD) scores used as main exposures in this study | 16-20 |

| **Supplementary Table 1.** Characteristics of 2060 included children and 4810 excluded children and their mothers. | | | | | |
| --- | --- | --- | --- | --- | --- |
|  | **2060 included** |  | **4810 excluded** |  |  |
|  | n (%) | Missing, n | n (%) | Missing, n | P |
| **Child characteristics** | 2060 |  | 4810 |  |  |
| Girls | 1023 (49.7) | - | 2317 (48.2) | - | 0.258^a^ |
| Cesarean section delivery | 360 (17.5) | 3 (0.1) | 872 (18.1) | 73 (1.5) | 0.525^a^ |
| Gestational age (weeks, mean (SD)) | 39.4 (1.6) | - | 39.2 (1.88) | - | <0.00^b^ |
| Birthweight (grams, mean (SD)) | 3557 (509) | 4 (0.0) | 3517 (559) | 77 (1.6) | 0.006^b^ |
| Type of feeding at 4 months of age |  | 55 (2.7) |  | 1304 (27.1) |  |
| Exclusive breastfeeding | 1339 (65.0) |  | 2317 (48.2) |  | 0.725^a^ |
| Partially breastfed | 324 (15.7) |  | 561 (11.7) |  |  |
| Exclusive formula feeding | 342 (16.6) |  | 628 (13.1) |  |  |
| Any breastfeeding at 9 months of age | 809 (39.3) | 65 (3.2) | 1091 (22.7) | 2057 (42.8) | 0.522^a^ |
| Adverse events from food 9 months | 271 (13.1) | 1 (0.0) | 346 (7.2) | 1984 (41.2) | 0.340^a^ |
| FA diagnosis at 9 months of age | 73 (3.5) | 1 (0.0) | 103 (3.6) | 1984 (41.2) | 0.854^a^ |
| FA diagnosis at 18 months of age | 100 (4.9) | - | 59 (4.7) | 3544 (73.7) | 0.799 |
| Family history of FA | 564 (27.4) | 26 (1.3) | 1062 (22.1) | 253 (5.3) | <0.001^a^ |
| Study Site |  | - |  | - | <0.001^a^ |
| Umeå | 1703 (82.7) |  | 3709 (77.1) |  |  |
| Skellefteå | 357 (17.3) |  | 1006 (20.9) |  |  |
| Lycksele ^d^ | 0 (0.0) |  | 95 (2.0) |  |  |
| Outdoor environment |  | - |  | 4 (0.1 | 0.003^a^ |
| City | 1261 (61.2) |  | 2984 (62.0) |  |  |
| Community | 392 (19.0) |  | 1024 (21.3) |  |  |
| Rural | 407 (19.8) |  | 798 (16.6) |  |  |
|  |  |  |  |  |  |
| **Maternal characteristics** | **2047^c^ included** |  | **4748 excluded** |  |  |
| Country of origin other than Sweden | 158 (7.7) | 43 (2.1) | 458 (9.6) | 397 (8.4) | <0.001^a^ |
| Maternal University education | 1420 (68.4) | 43 (2.1) | 2808 (59.1) | 407 (8.6) | <0.001^a^ |
| Maternal history of FA | 363 (17.7) | 46 (2.2) | 644 (13.6) | 424 (8.9) | <0.001^a^ |
| BMI at registration MCC, mean (SD) | 25.00 (4.72) | 45 (2.2) | 25.1 (4.74) | 164 (3.5) | 0.408^b^ |
| Smoking status |  | 28 (1.4) |  | 445 (9.4) | 0.088^a^ |
| No smoking | 1959 (95.7) |  | 4125 (86.9) |  |  |
| Smoked within a month before pregnancy | 39 (1.9) |  | 107 (2.3) |  |  |
| Smoking in pregnancy | 21 (1.0) |  | 68 (1.4) |  |  |
| Maternal age at delivery in years, mean (SD) | 31.3 (4.3) | - | 30.8 (4.5) | - | <0.001^b^ |
| First-time mother | 1058 (51.7) | 3 (0.1) | 2111 (44.5) | 72 (1.5) | <0.001^a^ |
| ^a^ P from Chi-square test  ^b^ P from T-test  ^c^ 13 births were twin births  ^d^ Recruitment in Lycksele started in late 2021 why no participants were included in the present study  FA, food allergy; n, number; MHC, Maternity Care Center | | | | | |

| Supplementary Table 2. Comparison of mean (SD) values for each investigated diet diversity score between the group of infants with parentally reported early introduction (<4 months of age) to solid foods (n=264) and the group that had not introduced solid foods before the age of 4 months (n=1792). | | | |
| --- | --- | --- | --- |
|  |  | | |
| Diet diversity scores | **Early introduction** | **No early introduction** | ***P* ANOVA** |
|  | n=264 | n=1792 |  |
| Weighted diet diversity score at age 9 months | 20.27 (3.74) | 19.70 (3.94) | 0.027 |
| Diversity of introduced foods at age 6 months | 10.33 (2.17) | 8.79 (2.84) | <0.001 |
| Diversity of introduced foods at age 9 months | 11.69 (1.42) | 11.50 (1.53) | 0.060 |
| Diversity of introduced allergenic foods at age 6 months | 3.59 (1.26) | 2.84 (1.44) | <0.001 |
| Diversity of introduced allergenic foods at age 9 months | 4.23 (1.00) | 4.12 (1.07) | 0.096 |
| ANOVA, analysis of variance; SD, standard deviation |  |  |  |

| Supplementary Table 3. Food allergen exposure by age 18 months among children with no parentally reported food allergy at that age (N=1960) | | |
| --- | --- | --- |
| Number of allergens introduced by age 18 months | **N (%)** | **Cumulative %** |
| 0-3 allergens | 21 (1.1) | 1.1 |
| 4 allergens | 269 (13.7) | 14.8 |
| 5 allergens | 865 (44.1) | 58.9 |
| 6 allergens | 805 (41.1) | 100.0 |

| Supplementary Table 4. Different measures of diet diversity of complementary feeding in infancy and the association to food allergy at age 9 months presenting unadjusted and multivariable adjusted odds ratios (ORs) and 95% Confidence Intervals (CIs), together with P-values. | | | | | | | | | | | | |
| --- | --- | --- | --- | --- | --- | --- | --- | --- | --- | --- | --- | --- |
|  | **Unadjusted Model** | | | | **Multivariable model 1 ^d^** | | | | **Multivariable model 2 ^e^** | | | |
| Weighted diet diversity score at age 9 months ^a^ | **n, all** | **n, FA** | **OR (95% CI)** | **P** | **n, all** | **n, FA** | **OR (95% CI)** | **P** | **n, all** | **n, FA** | **OR (95% CI)** | **P** |
| Continuous, 0-31p | 2059 | 73 | 1.01 (0.95-1.07 | 0.765 | 1960 | 68 | 1.00 (0.94-1-07) | 0.905 | 1957 | 68 | 1.00 (0.94-1.07) | 0.952 |
| 0-17p | 494 | 15 | Ref 1.0 |  | 469 | 15 | Ref 1.0 |  | 469 | 15 | Ref 1.0 | Ref |
| 18-20p | 654 | 27 | 1.38 (0.72-2.61) | 0.331 | 622 | 25 | 1.30 (0.68-2.50) | 0.433 | 620 | 25 | 1.33 (0.69-2.56) | 0.401 |
| 21-23p | 582 | 23 | 1,31 (0.68-2.55) | 0.419 | 552 | 21 | 1.24 (0.63-2.45) | 0.534 | 552 | 21 | 1.20 (0.61-2.38) | 0.598 |
| 24-31p | 329 | 8 | 0.80 (0.33-.1.90) | 0.607 | 317 | 7 | 0.72 (0.29-1.80) | 0.481 | 316 | 7 | 0.70 (0.28-1.75) | 0.445 |
| Diversity of introduced foods at age 6 months ^b^ | |  |  |  |  |  |  |  |  |  |  |  |
| Continuous, 0-14 foods | 2055 | 73 | 1.01 (0.93-1.10) | 0.747 | 1960 | 68 | 1.00 (0.93-1.09) | 0.901 | 1957 | 68 | 1.01 (0.92-1.10) | 0.833 |
| 0-7 foods | 511 | 21 | Ref 1.0 |  | 485 | 19 | Ref 1.0 |  | 485 | 19 | Ref 1.0 |  |
| 8-9 foods | 563 | 15 | 1.25 (0.61-2.58) | 0.540 | 535 | 14 | 0.68 (0.34-1.38) | 0.284 | 534 | 14 | 0.68 (0.33-1.38) | 0.677 |
| 10-11 foods | 618 | 25 | 0.80 (0.37-1.73) | 0.572 | 597 | 25 | 1.03 (0.56-1.92) | 0.923 | 596 | 25 | 1.01 (0.54-1.89) | 0.970 |
| 12-14 foods | 363 | 12 | 1.23 (0-61-2.49) | 0.558 | 343 | 10 | 0.76 (0.34-1.69) | 0.507 | 342 | 10 | 0.71 (0.32-1.58) | 0.401 |
| Diversity of introduced foods at age 9 months ^b^ |  |  |  |  |  |  |  |  |  |  |  |  |
| Continuous, 0-14 foods | 2055 | 73 | 0.97 (0.83-1.12) | 0.673 | 1960 | 68 | 0.96 (0.81-1.12) | 0.569 | 1957 | 68 | 0.95 (0.81-1.11) | 0.495 |
| 0-10 foods | 465 | 18 | Ref 1.0 |  | 439 | 18 | Ref 1.0 |  | 438 | 18 | Ref 1.0 |  |
| 11 foods | 490 | 16 | 0.84 (0.42-1.66) | 0.614 | 465 | 15 | 0.73 (0.36-1.47) | 0.379 | 464 | 15 | 0.70 (0.35-1.43) | 0.329 |
| 12 foods | 518 | 23 | 1.15 (0.62-2.17) | 0.656 | 495 | 20 | 1.00 (0.52-1.93 | 0.998 | 495 | 20 | 0.97 (0.50-1.86) | 0.915 |
| 13-14 foods | 582 | 16 | 0.70 (0.35-1.39) | 0.311 | 561 | 15 | 0.66 (0.33-1.34) | 0.248 | 560 | 15 | 0.62 (0.31-1.26) | 0.186 |
| Diversity of allergenic foods introduced at age 6 months ^c^ | |  |  |  |  |  |  |  |  |  |  |  |
| Continuous, 0-6 allergenic foods | 2055 | 73 | 1.00 (0.85-1.18) | 0.964 | 1960 | 68 | 1.00 (0.84-1.20 | 0.962 | 1957 | 68 | 0.99 (0.83-1.18) | 0.897 |
| 0-2 allergenic foods | 776 | 26 | Ref 1.0 |  | 738 | 24 | Ref 1.0 |  | 738 | 24 | Ref 1.0 |  |
| 3 allergenic foods | 541 | 22 | 1.22 (0.69-2.18) | 0.496 | 513 | 21 | 1.19 (0.65-2.19) | 0.566 | 512 | 21 | 1.18 (0.64-2.17) | 0.592 |
| 4 allergenic foods | 442 | 17 | 1.15 (0.62-2.15) | 0.652 | 426 | 16 | 1.14 (0.59-2.18) | 0.705 | 425 | 16 | 1.08 (0.56-2.09) | 0.810 |
| 5-6 allergenic foods | 296 | 8 | o.80 (0.36-1.79) | 0.589 | 283 | 7 | 0.77 (0.32-1.84) | 0.562 | 282 | 7 | 0.72 (0.30-1.74) | 0.470 |
| Diversity of allergenic foods introduced at age 9 months ^c^ | |  |  |  |  |  |  |  |  |  |  |  |
| Continuous, 0-6 allergenic foods | 2055 | 73 | 0.94 (0.76-1.17) | 0.591 | 1960 | 68 | 0.92 (0.73-1.16) | 0.472 | 1957 | 68 | 0.91 (0.72-1.14) | 0.412 |
| 0-3 allergenic foods | 548 | 18 | Ref 1.0 |  | 518 | 18 | Ref 1.0 |  | 517 | 18 | Ref 1.0 |  |
| 4 allergenic foods | 740 | 32 | 1.33 (0.74-2.40 | 0.341 | 703 | 29 | 1.19 (0.65-2.17) | 0.574 | 702 | 29 | 1.18 (0.64.2.16) | 0.597 |
| 5 allergenic foods | 575 | 18 | 0.95 (0.49-1.85) | 0.883 | 554 | 17 | 0.92 (0.47-1.82) | 0.817 | 554 | 17 | 0.88 (0.45-1.45) | 0.722 |
| 6 allergenic foods | 192 | 5 | 0.79 (0.29-2.15) | 0.641 | 185 | 4 | 0.65 (0.22-1.98) | 0.454 | 184 | 4 | 0.63 (0.51-1.92) | 0.417 |
| ^a^ Based on consumption frequency of 14 different food groups, each group contributing with 0-2 or 0-3 points to the total score.  ^b^ Based on introduction (yes/no) of 14 different food groups (potatoes, rice, pasta, vegetables, meat, fish, soy, legumes, fruit and berries, egg, dairy, porridge, bread, nuts & peanuts).  ^c^ Based on introduction (yes/no) of 6 different allergenic foods: milk, wheat, egg, fish, soy, nuts & peanuts.  ^d^ Adjusted for ethnicity (Swedish or other), maternal University education (yes/no), breastfeeding status at 4 months (exclusively breastmilk, breastmilk and formula, or only formula), and early (<4 months) introduction of solid foods (yes/no).  ^e^ Adjusted for ethnicity (Swedish or other), maternal University education (yes/no), breastfeeding status at 4 months  (exclusively breastmilk, breastmilk and formula, or only formula), early (< 4 months) introduction of solid foods (yes/no), FA in the family (mother, and/or father, and/or sibling), housing outdoor environment (city, smaller village, or rural), and maternal age at delivery.  CI, confidence interval; DD, diet diversity; FA, food allergy; N, number; OR, odds ratio; p, points | | | | | | | | | | | | |

| Supplementary Table 5. Sensitivity analysis for associations between diet diversity of complementary feeding in infancy and food allergy at age 18 months, excluding children with a food allergy diagnosis already at age 9 months (n=48), presenting unadjusted and multivariable adjusted odds ratios (ORs) and 95% Confidence Intervals (CIs), together with P-values. | | | | | | | | | | | | |
| --- | --- | --- | --- | --- | --- | --- | --- | --- | --- | --- | --- | --- |
|  | **Unadjusted Model** | | | | **Multivariable model 1 ^a^** | | | | **Multivariable model 2 ^b^** | | | |
| Weighted diet diversity score at age 9 months ^c^ | **n** | **FA cases** | **OR (95% CI)** | **P** | **n** | **FA cases** | **OR (95% CI)** | **P** | **n** | **FA cases** | **OR (95% CI)** | **P** |
| Continuous, 0-31p | 1986 | 52 (2.6) | 0.94 (0.88-1.01) | 0.069 | 1892 | 48 (2.5) |  | 0.067 | 1889 | 48 (2.5) | 0.94 (0.87-1.00) | 0.055 |
| 0-17p | 479 | 17 (3.5) | Ref 1.0 |  | 454 | 17 (3.7) | Ref 1.0 | Ref | 454 | 17 (3.7) | Ref 1.0 | Ref |
| 18-20p | 627 | 17 (2.7) | 0.76 (0.38-1.50) | 0.425 | 597 | 13 (2.2) | 0.59 (0.28-1.52) | 0.154 | 595 | 13 (2.2) | 0.57 (0.27-1.20) | 0.139 |
| 21-23p | 559 | 15 (2.7) | 0.75 (0.37-1.52) | 0.423 | 531 | 15 (2.8) | 0.79 (0.39-1.60) | 0.503 | 531 | 15 (2.8) | 0.75 (0.37-1.53) | 0.749 |
| 24-31p | 321 | 3 (0.9) | 0.26 (0.08-0.88) | 0.031 | 310 | 3 (1.0) | 0.26 (0.08-0.91) | 0.034 | 309 | 3 (1.0) | 0.24 (0.07-0.83) | 0.024 |
|  |  |  |  |  |  |  |  |  |  |  |  |  |
| Diversity of introduced foods at age 6 months ^d^ |  |  |  |  |  |  |  |  |  |  |  |  |
| Continuous, 0-14 foods | 1982 | 52 (2.6) | 1.00 (0.91-1.10) | 0.975 | 1892 | 48 (2.5) | 1.00 (0.90-1.11) | 0.994 | 1889 | 48 (2.5) | 0.99 (0.89-1.10) | 0.832 |
| 0-10 foods | 490 | 11 (2.2) | Ref 1.0 |  | 466 | 11 (2.4) | Ref 1.0 |  | 466 | 11 (2.4) | Ref 1.0 |  |
| 11 foods | 548 | 15 (2.8) | 1.23 (0.56-2.69) | 0.613 | 521 | 13 (2.4) | 1.10 (0.49-2.50) | 0.813 | 520 | 13 (2.5) | 1.06 (0.47-2.39) | 0.899 |
| 12 foods | 593 | 16 (2.7) | 1.21 (0.56-2.63) | 0.634 | 572 | 15 (2.6) | 1.14 (0.51-2.52) | 0.749 | 571 | 15 (2.6) | 1.07 (0.48-2.38) | 0.866 |
| 13-14 foods | 351 | 10 (2.8) | 1.28 (0.54-3.04) | 0.581 | 333 | 9 (2.7) | 1.12 (0.50-3.05) | 0.655 | 332 | 9 (2.7) | 1.13 (0.46-2.81) | 0.790 |
|  |  |  |  |  |  |  |  |  |  |  |  |  |
| Diversity of introduced foods at age 9 months ^d^ |  |  |  |  |  |  |  |  |  |  |  |  |
| Continuous, 0-14 foods | 1982 | 52 (2.6) | 0.85 (0.72-1.01) | 0.058 | 1892 | 48 (2.5) | 0.88 (0.73-1.05) | 0.141 | 1889 | 48 (2.5) | 0.87 (0.73-1.03) | 0.110 |
| 0-10 foods | 447 | 18 (4.0) | Ref 1.0 |  | 421 | 15 (3.6) | Ref 1.0 |  | 420 | 15 (3.6) | Ref 1.0 |  |
| 11 foods | 474 | 12 (2.5) | 0.62 (0.30-1.30) | 0.083 | 450 | 12 (2.7) | 0.73 (0.34-1.58) | 0.423 | 449 | 12 (2.7) | 0.72 (0.33-1.57) | 0.413 |
| 12 foods | 495 | 11 (2.2) | 0.54 (0.25-1.16) | 0.114 | 475 | 10 (2.1) | 0.61 (0.27-1.37) | 0.227 | 475 | 10 (2.1) | 0.58 (0.26-1.32) | 0.197 |
| 13-14 foods | 566 | 11 (1.9) | 0.47 (0.22-1.01) | 0.053 | 546 | 11 (2.0) | 0.59 (0.27-1.29) | 0.184 | 545 | 11 (2.0) | 0.55 (0.25-1.22) | 0.139 |
|  |  |  |  |  |  |  |  |  |  |  |  |  |
| Diversity of allergenic foods introduced at age 6 months ^e^ |  |  |  |  |  |  |  |  |  |  |  |  |
| Continuous, 0-6 allergenic foods | 1982 | 52 (2.6) | 1.07 (0.89-1.30) | 0.482 | 1892 | 48 (2.5) | 1.09 (0.89-1.33) | 0.422 | 1889 | 48 (2.5) | 1.07 (0.87-1.31) | 0.534 |
| 0-2 allergenic foods | 750 | 15 (2.0) | Ref 1.0 |  | 714 | 14 (2.0) | Ref 1.0 |  | 714 | 14 (2.0) | Ref 1.0 |  |
| 3 allergenic foods | 519 | 17 (3.3) | 1.66 (0.82-3.53) | 0.158 | 492 | 15 (3.0) | 1.59 (0.76-3.33) | 0.224 | 491 | 15 (3.1) | 1.53 (0.73-3.23) | 0.260 |
| 4 allergenic foods | 425 | 12 (2.8) | 1.42 (0.66-3.07) | 0.368 | 410 | 11 (2.7) | 1.44 (0.64-3.21) | 0.380 | 409 | 11 (2.7) | 1.36 (0.81-3.05) | 0.458 |
| 5-6 allergenic foods | 288 | 8 (2.8) | 1.40 (0.59-3.34) | 0.448 | 276 | 8 (2.9) | 1.58 (0.65-3.85) | 0.316 | 275 | 8 (2,9) | 1.47 (0.60-3.60) | 0.398 |
|  |  |  |  |  |  |  |  |  |  |  |  |  |
| Diversity of allergenic foods introduced at age 9 months ^e^ |  |  |  |  |  |  |  |  |  |  |  |  |
| Continuous, 0-6 allergenic foods | 1982 | 52 (2.6) | 0.92 (0.71-1.18) | 0.500 | 1892 | 48 (2.5) | 0.96 (0.74-1.26) | 0.789 | 1889 | 48 (2.5) | 0.95 (0.72-1.25) | 0.707 |
| 0-3 allergenic foods | 530 | 15 (2.8) | Ref 1.0 |  | 500 | 13 (2.6) | Ref 1.0 |  | 499 | 13 (2.6) | Ref 1.0 |  |
| 4 allergenic foods | 708 | 20 (2.8) | 1.00 (0.51-1.97) | 0.996 | 674 | 18 (2.7) | 1.03(0.50-2.13) | 0.932 | 673 | 18 (2.7) | 1.03 (0.50-2.14) | 0.928 |
| 5 allergenic foods | 557 | 13 (2.3) | 0.89 (0.42-1.85) | 0.746 | 537 | 14 (2.6) | 1.05 (0.49-2.27) | 0.894 | 537 | 14 (2.6) | 1.02 (0.47-2.20) | 0.966 |
| 6 allergenic foods | 187 | 3 (1.6) | 0.56 (0.16-1.96) | 0.363 | 181 | 3 (1.7) | 0.67 (0.19-2.38 | 0.536 | 180 | 3 (1.7) | 0.63 (0.18-2.27) | 0.484 |
|  |  |  |  |  |  |  |  |  |  |  |  |  |
| ^a^ Adjusted for ethnicity (Swedish or other), maternal University education (yes/no), breastfeeding status at 4 months (exclusively breastmilk, breastmilk and formula, or only formula), and early (<4 months age) introduction of solid foods (yes/no)  ^b^  Adjusted for ethnicity (Swedish or other), maternal University education (yes/no), breastfeeding status at 4 months (exclusively breastmilk, breastmilk and formula, or only formula), early (< 4 months age) introduction of solid foods (yes/no), FA in the family (mother, and/or father, and/or sibling), outdoor living environment (City, smaller village, or rural), and maternal age at delivery  ^c^ Based on the frequency of consumption of 14 different food groups (potato, rice, pasta, pulses, meat, fish, egg, dairy, porridge, bread, nuts and peanuts, vegetables, vegetarian meat substitutes, fruit and berries), each group contributing with 0-2 or 0-3 points to the total score  ^d^ Based on intake (yes/no) of 14 different food groups (potatoes, rice, pasta, vegetables, meat, fish, soy, legumes, fruit and berries, egg, dairy, porridge, bread, nuts and  ^e^ Based on intake (yes/no) of 6 different allergenic foods: milk, wheat, egg, fish, soy, nuts and peanuts  CI, confidence interval; DD, diet diversity; FA, food allergy; N, number; OR, odds ratio; p. points | | | | | | | | | | | | |

| Supplementary Table 6. Sensitivity analysis for the association between diet diversity of complementary feeding in infancy and food allergy (FA) at age 18 months, excluding children with reported gastrointestinal symptoms as response to their FA (n=33), presenting unadjusted and multivariable adjusted odds ratios (ORs) and 95% Confidence Intervals (CIs), together with P-values. | | | | | | | | | | | | |
| --- | --- | --- | --- | --- | --- | --- | --- | --- | --- | --- | --- | --- |
|  | | | | | | | | | | | | |
|  | **Unadjusted Model** | | | | **Multivariable model 1 ^a^** | | | | **Multivariable model 2 ^b^** | | | |
| Weighted diet diversity score at age 9 months ^c^ | **n** | **FA cases** | **OR (95% CI)** | **P** | **n** | **FA cases** | **OR (95% CI)** | **P** | **n** | **FA cases** | **OR (95% CI)** | **P** |
| Continuous 0-31p | 2027 | 67 (3.3) | 0.96 (0.90-1.02)) | 0.164 | 1931 | 63 (3.3) | 0.95 (0.90-1.02) | 0.136 | 1928 | 63 (3.3) | 0.95 (0.89-1.01) | 0.106 |
| 0-17p | 485 | 19 (3.9) | Ref 1.0 |  | 460 | 19 (4.1) | Ref 1.0 | Ref | 469 | 19 (4.1) | Ref 1.0 | Ref |
| 18-20p | 645 | 25 (3.9) | 0.99 (0.54-1.82) | 0.971 | 616 | 23 (3.7) | 0.92 (0.49-1.71) | 0.784 | 620 | 23 (3.7) | 0.90 (0.48-1.69) | 0.749 |
| 21-23p | 571 | 18 (3.2) | 0.80 (0.41-1.54) | 0.501 | 542 | 17 (3.1) | 0.79 (0.40-1.54) | 0.486 | 542 | 17 (3.1) | 0.76 (0.39-1.50) | 0.432 |
| 24-31p | 326 | 5 (1.5) | 0.38 (0.214-1.03) | 0.058 | 313 | 4 1.3) | 0.31 (0.10-0.92) | 0.035 | 313 | 4 (1.3) | 0.29 (0.10-0.88) | 0.028 |
|  |  |  |  |  |  |  |  |  |  |  |  |  |
| Diversity of introduced foods at age 6 months ^d^ |  |  |  |  |  |  |  |  |  |  |  |  |
| Continuous, 0-14 foods | 2023 | 67 (3.3) | 1.00 (0.92-1.09) | 0.997 | 1931 | 63 (3.3) | 0.98 (0.90-1.07) | 0.678 | 1928 | 63 (3.3) | 0.97 (0.88-1.06) | 0.498 |
| 0-7 foods | 503 | 15 (3.0) | Ref 1.0 |  | 477 | 15 (3.1) | Ref 1.0 |  | 477 | 15 (3.1) | Ref 1.0 |  |
| 8-9 foods | 555 | 16 (2.9) | 1.05 (0.47-2.38) | 0.898 | 529 | 15 (2.8) | 0.92 (0.44-1.92) | 0.826 | 528 | 15 2.8) | 0.87 (0.42-1.82) | 0.717 |
| 10-11 foods | 612 | 26 (4.2) | 1.02 (0.46-2.27) | 0.965 | 591 | 25 (4.2) | 1.30 (0.67-2.52 | 0.434 | 590 | 25 (4.2) | 1.22 (0.63-2.38 | 0.558 |
| 12-14 foods | 353 | 10 (2.8) | 1.52 (0.73-3.19) | 0.267 | 334 | 8 (2.4) | 0.77 (0.32-1.87) | 0.565 | 333 | 8 (2.4) | 0.71 (0.29-1.73) | 0.446 |
|  |  |  |  |  |  |  |  |  |  |  |  |  |
| Diversity of introduced foods at age 9 months ^d^ |  |  |  |  |  |  |  |  |  |  |  |  |
| Continuous, 0-14 foods | 2023 | 67 (3.3) | 0.84 (0.73-0.97) | 0.017 | 1931 | 63 (3.3) | 0.83 (0.71-0.97) | 0.020 | 1928 | 63 (3.3) | 0.82 (0.71-0.96) | 0.014 |
| 1-10 foods | 456 | 23 (5.0) | Ref 1.0 |  | 431 | 21 (4.9) | Ref 1.0 |  | 430 | 21 (4.9) | Ref 1.0 |  |
| 11 foods | 486 | 17 (3.5) | 0.68 (0.36-1.30) | 0.682 | 462 | 17 (3.7) | 0.71 (0.37-1.36) | 0.299 | 461 | 17 (3.7) | 0.70 (0.36-1.36) | 0.295 |
| 12 foods | 509 | 14 (2.8) | 0.53 (0.27-1.05) | 0.068 | 487 | 13 (2.7) | 0.55 (0.27-1.11) | 0.095 | 487 | 13 (2.7) | 0.52(0.26-1.07) | 0.076 |
| 13-14 foods | 572 | 13 (2.3) | 0.44 (0.22-0.87) | 0.019 | 551 | 12 (2.2) | 0.45 (0.22-0.92) | 0.030 | 550 | 12 (2.2) | 0.42 (0.20-0.88) | 0.021 |
|  |  |  |  |  |  |  |  |  |  |  |  |  |
| Diversity of allergenic foods introduced at age 6 months ^e^ |  |  |  |  |  |  |  |  |  |  |  |  |
| Continuous, 0-6 allergenic foods | 2023 | 67 (3.3) | 1.06 (0.90-1.26) | 0.472 | 1931 | 63 (3.3) | 1.04 (0.87-1.25) | 0.673 | 1928 | 63 (3.3) | 1.02 (0.85-1.23) | 0.833 |
| 0-2 allergenic foods | 763 | 15 (2.0) | Ref 1.0 |  | 726 | 15 (2.1) | Ref 1.0 |  | 726 | 15 (2.1) | Ref 1.0 |  |
| 3 allergenic foods | 536 | 29 (5.4) | 2.85 (1.51-5-37) | 0.001 | 509 | 27 (5.3) | 2.54 (1.33-4.87) | 0.005 | 508 | 27 (5.3) | 2.44 (1.27-4.69) | 0.007 |
| 4 allergenic foods | 432 | 14 (3.2) | 1.67 (0.80-3.49) | 0.173 | 417 | 13 (3.1) | 1.50 (0.70-3.22) | 0.293 | 416 | 13 (3.1) | 1.45 (0.67-3.10) | 0.345 |
| 5-6 allergenic foods | 292 | 9 (3.1) | 1.59 (0.69-3.67) | 0.281 | 279 | 8 (2.9) | 1.43 (0.59-3.46) | 0.427 | 278 | 8 (2.9) | 1.33 (0.55-3.22) | 0.535 |
|  |  |  |  |  |  |  |  |  |  |  |  |  |
| Diversity of allergenic foods introduced at age 9 months ^e^ |  |  |  |  |  |  |  |  |  |  |  |  |
| Continuous, 0-6 allergenic foods | 2023 | 67 (3.3) | 0.82 (0.65-1.03) | 0.083 | 1931 | 63 | 0.82 (0.65-1.04) | 0.099 | 1928 | 63 | 0.81 (0.64-1.03) | 0.079 |
| 0-3 allergenic foods | 542 | 23 | Ref 1.0 |  | 518 | 22 | Ref 1.0 |  | 512 | 22 | Ref 1.0 |  |
| 4 allergenic foods | 727 | 24 | 0.77 (0.43-1.38) | 0.380 | 703 | 22 | 0.73 (0.40-1.34) | 0.305 | 691 | 22 | 0.73 (0.40-1.35) | 0.317 |
| 5 allergenic foods | 265 | 18 | 0.74 (0.40-1.39) | 0.353 | 554 | 17 | 0.75 (0.39-1.44) | 0.391 | 544 | 17 | 0.72 (0.37-1.38) | 0.322 |
| 6 allergenic foods | 189 | 2 | 0.24 (0.06-1.03) | 0.055 | 185 | 2 | 0.26 (0.06-1.13) | 0.072 | 181 | 2 | 0.24 (0.06-1.08) | 0.063 |
| ^a^ Adjusted for ethnicity (Swedish or other), maternal University education (yes/no), breastfeeding status at 4 months (exclusively breastmilk, breastmilk and formula, or only formula), and early (<4 months age) introduction of solid foods (yes/no)  ^b^  Adjusted for ethnicity (Swedish or other), maternal University education (yes/no), breastfeeding status at 4 months (exclusively breastmilk, breastmilk and formula, or only formula), early (< 4 months age) introduction of solid foods (yes/no), FA in the family (mother, and/or father, and/or sibling), outdoor living environment (city, smaller village, or rural), and maternal age at delivery  ^c^ Based on the frequency of consumption of 14 different food groups (potato, rice, pasta, pulses, meat, fish, egg, dairy, porridge, bread, nuts and peanuts, vegetables, vegetarian meat substitutes, fruit and berries), each group contributing with 0-2 or 0-3 points to the total score  ^d^ Based on intake (yes/no) of 14 different food groups (potatoes, rice, pasta, vegetables, meat, fish, soy, legumes, fruit and berries, egg, dairy, porridge, bread, nuts and peanuts)  ^e^ Based on intake (yes/no) of 6 different allergenic foods (milk, wheat, egg, fish, soy, nuts and peanuts)  CI, confidence interval; DD, diet diversity; FA, food allergy; N, number; OR, odds ratio; p, points | | | | | | | | | | | | |

| **Supplementary Table 7.** Risk estimates from the multivariable logistic regression model 2 for the association between different measures of diet diversity of complementary foods at 6- and 9-months age and the risk of food allergy (FA) at age 18 months, presenting odds ratios (ORs) and 95% Confidence Intervals (CIs), together with P-values. | | | |
| --- | --- | --- | --- |
|  | **n all/cases** | **OR (95% CI)** | **P** |
| **Weighted diet diversity score at age 9 months ^a^** | 1957/92 |  |  |
| Continuous, 0-31 points |  | 0.96 (0.91-1.01) | 0.128 |
| Ethnicity (Swedish vs other) |  | 0.26 (0.06-1.05) | 0.059 |
| Maternal education (university vs lower) |  | 1.14 (0.69-1.91) | 0.604 |
| Breastfeeding at 4 months |  |  |  |
| Exclusive breastfeeding |  | Ref 1.0 |  |
| Breastmilk and formula feeding |  | 1.77 (1.03-3.05) | 0.038 |
| Only formula feeding |  | 1.89 (1.12-3.18) | 0.017 |
| Early introduction of solid foods (<4 months) |  | 1.26 (0.70-2.25) | 0.439 |
| Outdoor living environment |  |  |  |
| City/Urban |  | Ref 1.0 |  |
| Smaller village |  | 1.10 (0.66-1.85) | 0.712 |
| Rural |  | 0.55 (0.27-1.10) | 0.089 |
| FA in the family |  | 2.51 (1.64-3.84) | <0.001 |
| Maternal age at delivery (years) |  | 0.99 (0.94-1.04) | 0.673 |
|  |  |  |  |
| **Diversity of introduced foods at age 6 months ^b^** | 1957/92 |  |  |
| Continuous, 0-14 different foods |  | 0.99 (0.92-1.08) | 0.885 |
| Ethnicity (Swedish vs other) |  | 0.25 (0.06-1.04) | 0.056 |
| Maternal education (university vs lower) |  | 1.14 (0.69-1.89) | 0.617 |
| Breastfeeding at 4 months |  |  |  |
| Exclusive breastfeeding |  | Ref 1.0 |  |
| Breastmilk and formula feeding |  | 1.77 (1.04-3.08) | 0.035 |
| Only formula feeding |  | 1.90 (1.13-3.19) | 0.016 |
| Early introduction of solid foods (<4 months) |  | 1.24 (0.68-2.24) | 0.483 |
| Outdoor living environment |  |  |  |
| City/Urban |  | Ref 1.0 |  |
| Smaller village |  | 1.08 (0.65-1.82) | 0.758 |
| Rural |  | 0.56 (0.28-1.12) | 0.099 |
| FA in the family |  | 2.49 (1.63-3.82) | <0.001 |
| Maternal age at delivery (years) |  | 0.99 (0.94-1.04) | 0.676 |
|  |  |  |  |
| **Diversity of introduced foods at age 9 months ^b^** | 1957/92 |  |  |
| Continuous, 0-14 different foods |  | 0.88 (0.77-1.01) | 0.065 |
| Ethnicity (Swedish vs other) |  | 0.26 (0.06-1.09) | 0.065 |
| Maternal education (University vs lower) |  | 1.15 (0.69-1.92) | 0.588 |
| Breastfeeding at 4 months |  |  |  |
| Exclusive breastfeeding |  | Ref 1.0 |  |
| Breastmilk and formula feeding |  | 1.78 (1.03-3.06) | 0.037 |
| Only formula feeding |  | 1.92 (1.14-3.23) | 0.014 |
| Early introduction of solid foods (<4 months) |  | 1.25 (0.70-2.24) | 0.452 |
| Outdoor living environment |  |  |  |
| City/Urban |  | Ref 1.0 |  |
| Smaller village |  | 1.11 (0.66-1.86) | 0.691 |
| Rural |  | 0.56 (0.28-1.12) | 0.099 |
| FA in the family |  | 2.53 (1.65-3.87) | <0.001 |
| Maternal age at delivery (years) |  | 0.99 (0.94-1.04) | 0.676 |
|  |  |  |  |
| **Diversity of introduced allergenic foods at age 6 months ^c^** | 1957/92 |  |  |
| Continuous, 0-6 different foods |  | 1.03 (0.89-1.21) | 0.667 |
| Ethnicity (Swedish vs other) |  | 0.25 (0.06-1.03) | 0.055 |
| Maternal education (university vs lower) |  | 1.13 (0.68-1.89) | 0.631 |
| Breastfeeding at 4 months |  |  |  |
| Exclusive breastfeeding |  | Ref 1.0 |  |
| Breastmilk and formula feeding |  | 1.80 (1.05-3.10) | 0.033 |
| Only formula feeding |  | 1.90 (1.13-3.20) | 0.015 |
| Early introduction of solid foods (<4 months) |  | 1.20 (0.66-2.16) | 0.551 |
| Outdoor living environment |  |  |  |
| City/Urban |  | Ref 1.0 |  |
| Smaller village |  | 1.08 (0.65-1.81) | 0.763 |
| Rural |  | 0.56 (0.28-1.12) | 0.100 |
| FA in the family |  | 2.47 (1.61-3.79) | <0.001 |
| Maternal age at delivery (years) |  | 0.99 (0.94-1.04) | 0.682 |
|  |  |  |  |
| **Diversity of introduced allergenic foods at age 9 months ^c^** | 1957/92 |  |  |
| Continuous, 0-6 different foods |  | 0.91 (0.75-1.12) | 0.371 |
| Ethnicity (Swedish vs other) |  | 0.26 (0.06-1.07) | 0.061 |
| Maternal education (university vs lower) |  | 1.14 (0.69-1.90) | 0.605 |
| Breastfeeding at 4 months |  |  |  |
| Exclusive breastfeeding |  | Ref 1.0 |  |
| Breastmilk and formula feeding |  | 1.80 (1.04-3.08) | 0.034 |
| Only formula feeding |  | 1.89 (1.13-3.19) | 0.016 |
| Early introduction of solid foods (<4 months) |  | 1.23 (0.69-2.21) | 0.479 |
| Outdoor living environment |  |  |  |
| City/Urban |  | Ref 1.0 |  |
| Smaller village |  | 1.10 (0.66-1.84) | 0.720 |
| Rural |  | 0.56 (0.28-1.12) | 0.100 |
| FA in the family |  | 2.50 (1.63-3.83) | <0.001 |
| Maternal age at delivery (years) |  | 0.99 (0.94-1.04) | 0.672 |
| ^a^ Based on consumption frequency of 14 different food groups, each group contributing with 0-2 or 0-3 points to the total score.  ^b^ Based on introduction (yes/no) of 14 different food groups (potatoes, rice, pasta, vegetables, meat, fish, soy, legumes, fruit and berries, egg, dairy, porridge, bread, nuts & peanuts).  ^c^ Based on introduction (yes/no) of 6 different allergenic foods: milk, wheat, egg, fish, soy, nuts & peanuts.  CI, confidence interval; DD, diet diversity; FA, food allergy; N, number; OR, odds ratio; p, points | | | |
| \| Supplementary Table 8. Stratified analyses of children without vs with a history of food allergy in the closest family (mother and/or father and/or sibling), estimating unadjusted and adjusted associated risk for the child developing their own food allergy at age 18 months in relation to two measurements of diet diversity at age 9 months (weighted diet diversity score and diversity of introduced foods). \| \| \| \| \| \| \| \| \| \| \| \| \| \| --- \| --- \| --- \| --- \| --- \| --- \| --- \| --- \| --- \| --- \| --- \| --- \| --- \| \|  \| **Unadjusted Model** \| \| \| \| **Multivariable model 1 ^a^** \| \| \| \| **Multivariable model 2 ^b^** \| \| \| \| \| Weighted diet diversity score at age 9 months ^c^ \| **n, all** \| **n, FA** \| **OR (95% CI)** \| **P** \| **n, all** \| **n, FA** \| **OR (95% CI)** \| **P** \| **n, all** \| **n, FA** \| **OR (95% CI)** \| **P** \| \|  \|  \|  \|  \|  \|  \|  \|  \|  \|  \|  \|  \|  \| \| NO food allergy in the family \|  \|  \|  \|  \|  \|  \|  \|  \|  \|  \|  \|  \| \| Continuous, 0-31p \| 1470 \| 52 \| 0.96 (0.89-1.02) \| 0.203 \| 1410 \| 48 \| 0.95 (0.88-1.02) \| 0.159 \| 1410 \| 48 \| 0.95 (0.88-1.02) \| 0.106 \| \| 0-17p \| 362 \| 17 \| Ref 1.0 \|  \| 344 \| 17 \| Ref 1.0 \| Ref \| 344 \| 17 \| Ref 1.0 \| Ref \| \| 18-20p \| 474 \| 17 \| 0.76 (0.38-1.50) \| 0.348 \| 455 \| 15 \| 0.66 (0.32-1.34) \| 0.248 \| 455 \| 15 \| 0.64 (0.32-1.32) \| 0.227 \| \| 21-23p \| 407 \| 14 \| 0.72 (0.35-1.49) \| 0.378 \| 391 \| 13 \| 0.70 (0.33-1.47) \| 0.344 \| 391 \| 13 \| 0.69 (0.33-1.45 \| 0.322 \| \| 24-31p \| 227 \| 4 \| 0.36 (0.12-1.10) \| 0.072 \| 220 \| 3 \| 0.29 (0.08-0.99) \| 0.048 \| 220 \| 3 \| 0.29 (0.08-0.99) \| 0.048 \| \| Food allergy in the family \|  \|  \|  \|  \|  \|  \|  \|  \|  \|  \|  \|  \| \| Continuous, 0-31p \| 564 \| 47 \| 0.98 (0.91-1.06) \| 0.635 \| 547 \| 44 \| 0.98 (0.90-1.05) \| 0.512 \| 547 \| 44 \| 0.97 (0.90-1.05) \| 0.439 \| \| 0-17p \| 128 \| 11 \| Ref 1.0 \|  \| 125 \| 11 \| Ref 1.0 \|  \| 125 \| 11 \| Ref 1.0 \|  \| \| 18-20p \| 170 \| 16 \| 1.11 (0.49-2.47) \| 0.808 \| 165 \| 14 \| 1.00 (0.43-2.31) \| 0.995 \| 165 \| 14 \| 0.99 (0.43-2.30) \| 0.978 \| \| 21-23p \| 168 \| 15 \| 1.04 (0.46-2.35) \| 0.920 \| 161 \| 14 \| 0.98 (0.43-2.26 \| 0.966 \| 161 \| 14 \| 0.96 (0.41-2.22 \| 0.917 \| \| 24-31p \| 98 \| 5 \| 0.57 (0.19-1.70) \| 0.316 \| 96 \| 5 \| 0.54 (0.18-1.63) \| 0.275 \| 96 \| 5 \| 0.52 (0.17-1.59) \| 0.253 \| \|  \|  \|  \|  \|  \|  \|  \|  \|  \|  \|  \|  \|  \| \| Diversity of introduced foods at age 9 months ^d^ \|  \|  \|  \|  \|  \|  \|  \|  \|  \|  \|  \|  \| \|  \|  \|  \|  \|  \|  \|  \|  \|  \|  \|  \|  \|  \| \| NO food allergy in the family \|  \|  \|  \|  \|  \|  \|  \|  \|  \|  \|  \|  \| \| Continuous, 0-14 foods \| 1466 \| 52 \| 0.87 (0.74-1.03) \| 0.100 \| 1410 \| 48 \| 0.86 (0.72-1.03) \| 0.093 \| 1410 \| 48 \| 0.86 (0.72-1.02) \| 0.089 \| \| 0-10 foods \| 346 \| 17 \| Ref 1.0 \|  \| 330 \| 16 \| Ref 1.0 \|  \| 330 \| 16 \| Ref 1.0 \|  \| \| 11 foods \| 351 \| 11 \| 0.63 (0.29-1.36) \| 0.235 \| 335 \| 11 \| 0.64 (0.29-1.40) \| 0.261 \| 335 \| 11 \| 0.63 (0.29-1.39) \| 0.255 \| \| 12 foods \| 362 \| 15 \| 0.84 (0.41-1.70) \| 0.623 \| 349 \| 13 \| 0.76 (0.36-1.61) \| 0.478 \| 349 \| 13 \| 0.76 (0.36-1.62) \| 0.478 \| \| 13-14 foods \| 407 \| 9 \| 0.44 (0.19-0.99) \| 0.049 \| 396 \| 8 \| 0.44 (0.18-1.04) \| 0.061 \| 396 \| 8 \| 0.44 (0.18-1.04) \| 0.060 \| \| Food allergy in the family \|  \|  \|  \|  \|  \|  \|  \|  \|  \|  \|  \|  \| \| Continuous, 0-14 foods \| 564 \| 47 \| 0.91 (0.75-1.11) \| 0.349 \| 547 \| 44 \| 0.91 (0.75-1.12) \| 0.383 \| 547 \| 44 \| 0.92 (0.75-1.13) \| 0.430 \| \| 0-10 foods \| 112 \| 14 \| Ref 1.0 \|  \| 108 \| 13 \| Ref 1.0 \|  \| 108 \| 13 \| Ref 1.0 \|  \| \| 11 foods \| 132 \| 10 \| 0.57 (0.24-1.35) \| 0.202 \| 129 \| 9 \| 0.53 (0.21-1.30) \| 0.166 \| 129 \| 9 \| 0.56 (0.23-1.41) \| 0.219 \| \| 12 foods \| 153 \| 9 \| 0.44 (0.18-1.05) \| 0.064 \| 146 \| 8 \| 0.44 (0.17-1.12) \| 0.085 \| 146 \| 8 \| 0.47 (0.18-1.20) \| 0.113 \| \| 13-14 foods \| 167 \| 14 \| 0.64 (0.29-1.40) \| 0.265 \| 164 \| 14 \| 0.65 (0.29-1.47) \| 0.301 \| 164 \| 14 \| 0.67 (0.30-1.51) \| 0.333 \| \| ^a^ Adjusted for ethnicity (Swedish or other), maternal University education (yes/no), breastfeeding status at 4 months (exclusively breastmilk, breastmilk and formula, or only formula), and early (<4 months) introduction of solid foods (yes/no)  ^b^  Adjusted for ethnicity (Swedish or other), maternal University education (yes/no), breastfeeding status at 4 months (exclusively breastmilk, breastmilk and formula, or only formula), early (< 4 months) introduction of solid foods (yes/no), FA in the family (mother, and/or father, and/or sibling), outdoor living environment (City, smaller village, or rural), and maternal age at delivery  ^c^ Based on the frequency of consumption of 14 different food groups (potato, rice, pasta, pulses, meat, fish, egg, dairy, porridge, bread, nuts and peanuts, vegetables, vegetarian meat substitutes, fruit and berries), each group contributing with 0-2 or 0-3 points to the total score  ^d^ Based on intake (yes/no) of 14 different food groups (potatoes, rice, pasta, vegetables, meat, fish, soy, legumes, fruit and berries, egg, dairy, porridge, bread, nuts and peanuts)  CI, confidence interval; DD, diet diversity; FA, food allergy; N, number; OR, odds ratio; p, points \| \| \| \| \| \| \| \| \| \| \| \| \| | | | |

| Supplementary Table 9. Stratified analyses of children without vs with a history of eczema up until 18 months of age, estimating unadjusted and adjusted associated odds for the child developing food allergy at age 18 months in relation to two measurements of diet diversity at 9 months of age (weighted diet diversity and diversity of introduced foods). | | | | | | | | | | | | |
| --- | --- | --- | --- | --- | --- | --- | --- | --- | --- | --- | --- | --- |
|  |  |  |  |  |  |  |  |  |  |  |  |  |
|  | **Unadjusted model** | |  |  | **Multivariable model 1 ^c^** | | |  | **Multivariable model 2 ^d^** | | |  |
| Weighted diet diversity score at age 9 months ^a^ | **n** | **FA cases** | **OR (95% CI)** | **P** | **n** | **FA cases** | **OR (95% CI)** | **P** | **n** | **FA cases** | **OR (95% CI)** | **P** |
| NO history of eczema by age 18 months | |  |  |  |  |  |  |  |  |  |  |  |
| Continuous, 0-31p | 1396 | 40 | 1.02 (0.94-1.11) | 0.668 | 1336 | 35 | 1.00 (0.92-1.10) | 0.925 | 1333 | 35 | 1.00 (0.92-1.10) | 0.963 |
| 0-17p | 341 | 6 | Ref 1.0 |  | 326 | 6 | Ref 1.0 | Ref | 326 | 6 | Ref 1.0 | Ref |
| 18-20p | 455 | 14 | 1.17 (0.50-2.74) | 0.716 | 434 | 13 | 0.88 (0.36-2.16) | 0.778 | 432 | 13 | 0.85 (0.34-2.11) | 0.723 |
| 21-23p | 395 | 10 | 1.26 (0.53-2.97) | 0.605 | 377 | 9 | 1.17 (0.49-2.84) | 0.722 | 377 | 9 | 1.14 (0.47-2.78) | 0.768 |
| 24-31p | 205 | 4 | 0.73 (0.22-2.42) | 0.611 | 199 | 3 | 0.50 (0.13-1.88) | 0.305 | 198 | 3 | 0.49 (0.13-1.85) | 0.291 |
| History of eczema by age 18 months |  |  |  |  |  |  |  |  |  |  |  |  |
| Continuous, 0-31p | 596 | 56 | 0.93 (0.87-0.99) | 0.029 | 565 | 54 | 0.94 84-1.00) | 0.069 | 565 | 54 | 0.93 (0.87-1.00) | 0.050 |
| 0-17p | 135 | 7 | Ref 1.0 |  | 128 | 7 | Ref 1.0 |  | 128 | 7 | Ref 1.0 |  |
| 18-20p | 180 | 12 | 0.68 (0.34-1.37) | 0.279 | 172 | 11 | 0.68 (0.32-1.43) | 0.306 | 172 | 11 | 0.66 (0.31-1.41) | 0.285 |
| 21-23p | 169 | 13 | 0.68 (0.33-1.39) | 0.29 | 159 | 12 | 0.75 (0.35-1.60) | 0.457 | 159 | 12 | 0.68 (0.31-1.47 | 0.325 |
| 24-31p | 112 | 4 | 0.30 (0.11-0.85) | 0.023 | 106 | 4 | 0.37 (0.13-1.05) | 0.062 | 106 | 4 | 0.34 (0.12-0.99) | 0.049 |
|  |  |  |  |  |  |  |  |  |  |  |  |  |
| Diversity of introduced foods at age 9 months ^b^ | |  |  |  |  |  |  |  |  |  |  |  |
| NO history of eczema by age 18 months | |  |  |  |  |  |  |  |  |  |  |  |
| Continuous, 0-14 foods | 1393 | 40 | 0.96 (0.78-1.18) | 0.683 | 1336 | 35 | 0.94 (0.76-1.17) | 0.581 | 1333 | 35 | 0.94 (0.76-1.16) | 0.558 |
| 0-10 foods | 325 | 8 | Ref 1.0 |  | 311 | 8 | Ref 1.0 |  | 310 | 8 | Ref 1.0 |  |
| 11 foods | 336 | 7 | 0.56 (0.22-1.43) | 0.222 | 316 | 6 | 0.54 (0.20-1.47) | 0.225 | 315 | 6 | 0.52 (0.19-1.43) | 0.203 |
| 12 foods | 352 | 12 | 0.84 (0.37-1.93) | 0.684 | 340 | 11 | 0.74 (0.30-1.82) | 0.514 | 340 | 11 | 0.73 (0.30-1.80) | 0.495 |
| 13-14 foods | 380 | 7 | 0.71 (0.30-1.65) | 0.422 | 369 | 6 | 0.67 (0.27-1.66) | 0.390' | 368 | 6 | 0.65 (0.26-1.61) | 0.353 |
| History of eczema by age 18 months |  |  |  |  |  |  |  |  |  |  |  |  |
| Continuous, 0-14 foods | 595 | 56 | 0.85 (0.71-1.00) | 0.054 | 565 | 54 | 0.87 (0.72-1.05) | 0.136 | 565 | 54 | 0.86 (0.71-1.04) | 0.112 |
| 0-10 foods | 121 | 7 | Ref 1.0 |  | 113 | 7 | Ref 1.0 |  | 113 | 7 | Ref 1.0 |  |
| 11 foods | 140 | 9 | 0.63 (0.29-1.35) | 0.232 | 138 | 9 | 0.56 (0.25-1.24) | 0.153 | 138 | 9 | 0.53 (0.24-1.21) | 0.132 |
| 12 foods | 155 | 11 | 0.56 (0.26-1.20) | 0.138 | 144 | 9 | 0.61 (0.27-1.39) | 0.240' | 144 | 9 | 0.58 (0.25-1.33) | 0.197 |
| 13-14 foods | 179 | 9 | 0.48 (0.22-1.03) | 0.059 | 170 | 9 | 0.57 (0.25-1.26) | 0.162 | 170 | 9 | 0.54 (0.24-1.22) | 0.136 |
| a Based on the frequency of consumption of 14 different food groups, each group contributing with 0-2 or 0-3 points to the total score  b Based on intake (yes/no) of 14 different food groups (potatoes, rice, pasta, vegetables, meat, fish, soy, legumes, fruit and berries, egg, dairy, porridge, bread, nuts & peanuts)  c Adjusted for ethnicity (Swedish or other), maternal University education (yes/no), breastfeeding status at 4 months (exclusively breastmilk, breastmilk and formula, or only formula), and early (<4 months) introduction of solid foods (yes/no)  d Adjusted for ethnicity (Swedish or other), maternal University education (yes/no), breastfeeding status at 4 months (exclusively breastmilk, breastmilk and formula, or only formula), early (< 4 months) introduction of solid foods (yes/no), FA in the family (mother, and/or father, and/or sibling), housing outdoor environment (City, smaller village, or rural), and maternal age at delivery  CI, confidence interval; DD, diet diversity; FA, food allergy; N, number; OR, odds ratio; p, points | | | | | | | | | | | | |


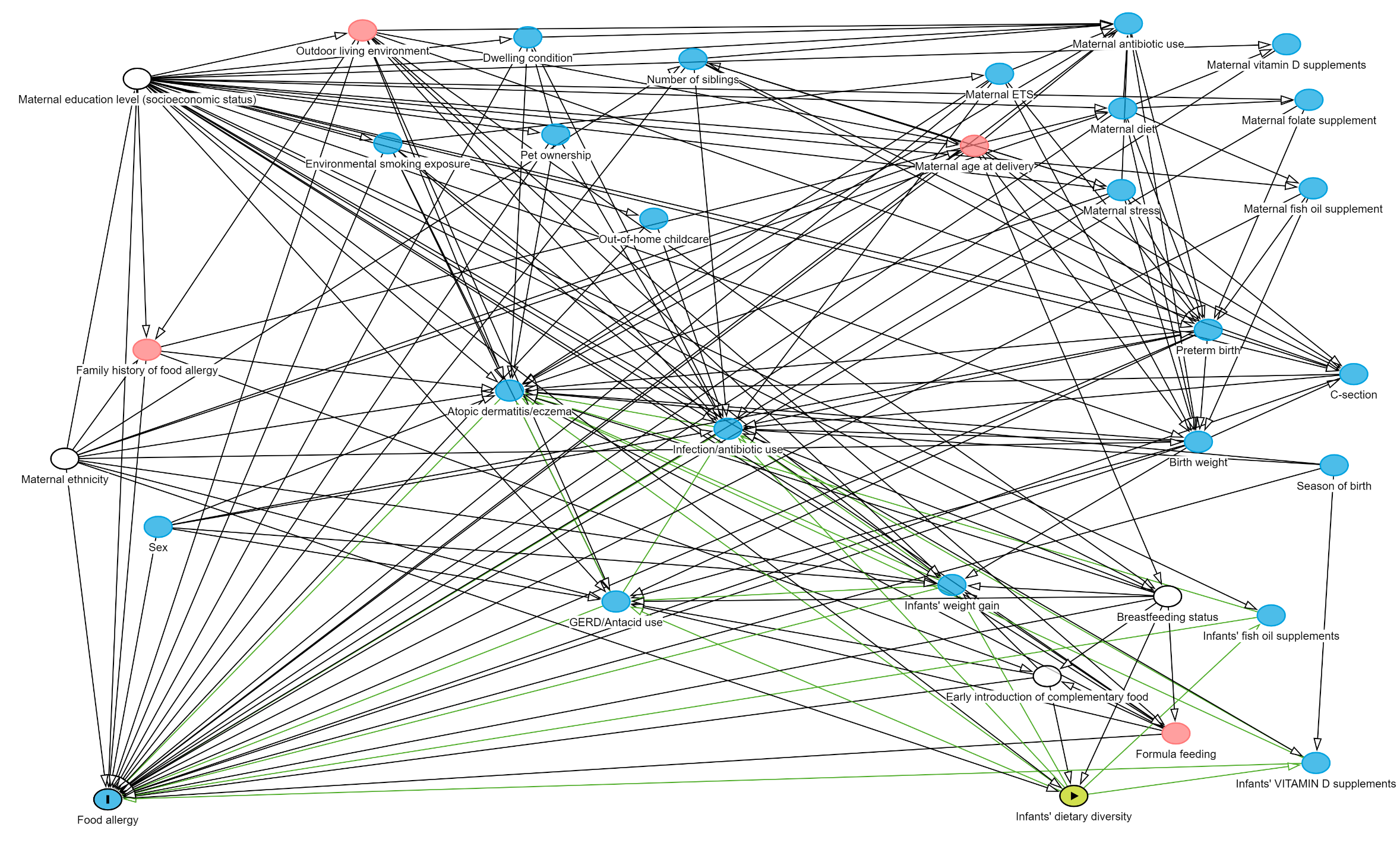


**Supplementary Figure 1.** Directed Acyclic Graph used for identification and selection of confounding factors used in multivariable model 1 and 2 for estimating associations between diet diversity and food allergy risk. Based on the DAGitty-code (<http://dagitty.net/dags.html?id=8J8AWP>) from Peng et al, Allergy 2024.

Food Allergy = Outcome

Infants' diet diversity = Main exposure

Covariates in white circles = Confounders adjusted for in multivariable model 1 in the main analysis in this study

Covariates in pink circles = Covariates obtained as ancestors of the outcome and the exposure and added as confounders in multivariable model 2 in the main analysis in this study

Covariates in blue circles = Other covariates obtained as ancestors of the outcome but not adjusted for in this study

**A)**


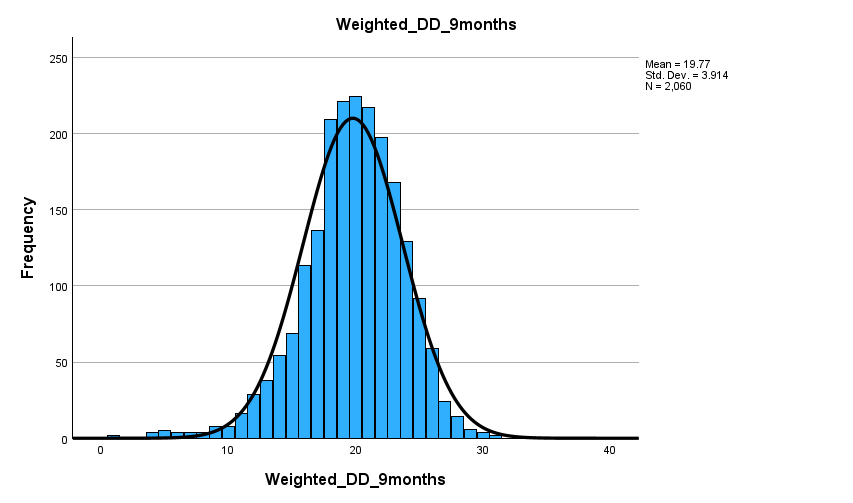


**B)**


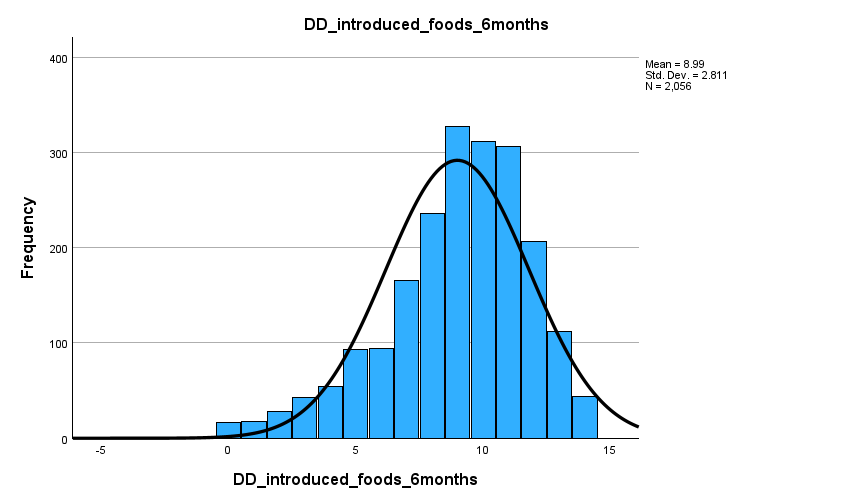


**C)**


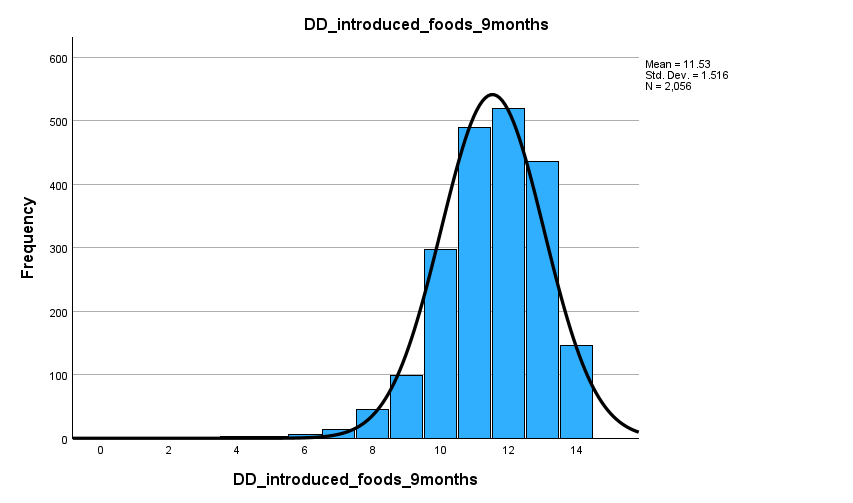


**D)**


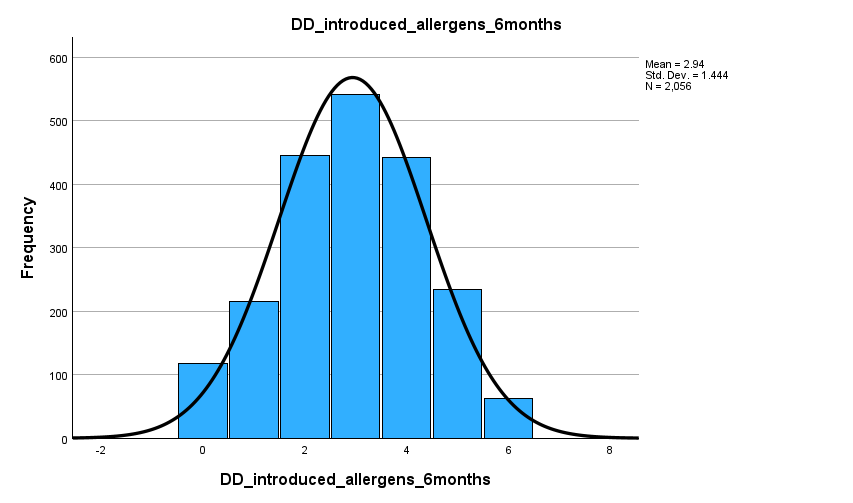


**E)**


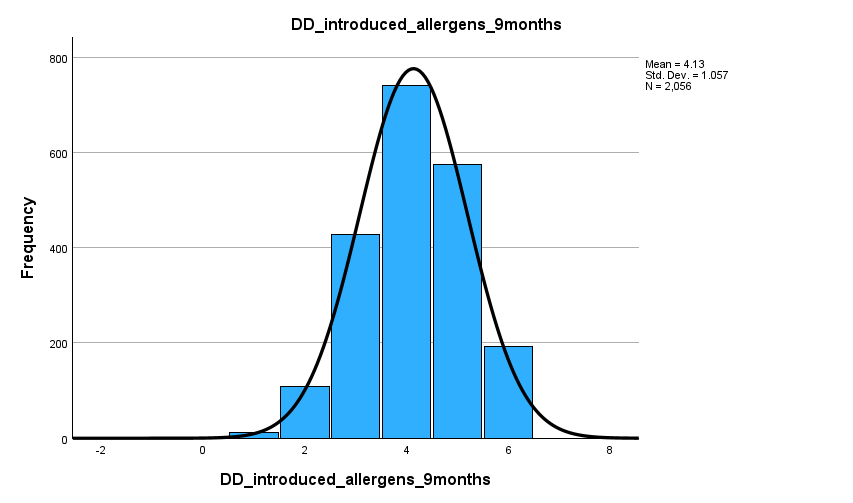


**Supplementary Figure 2.** Distribution and mean values (SD) of investigated diet diversity (DD) scores used as main exposures in this study for 2060 (A) or 2056 (B-E) participants for each investigated score; A) Weighted DD scores at age 9 months: B) Number of introduced foods at age 6 months; C) Number of introduced foods at age 9 months; D) Number of introduced allergenic foods at age 6 months; and E) Number of introduced allergenic foods at age 9 months. DD, diet diversity; SD, standard deviation
